# Comparative Chromatographic Analysis of Polyphenolic Compounds in Comfrey Leaf and Root with Determination of Their In Vitro Antioxidant and Anti-Inflammatory Activity

**DOI:** 10.3390/antiox15010046

**Published:** 2025-12-30

**Authors:** Katarzyna Kimel, Mirosława Krauze-Baranowska, Justyna Ośko, Małgorzata Grembecka, Barbara Sparzak-Stefanowska, Sylwia Godlewska

**Affiliations:** 1 Department of Pharmacognosy, Faculty of Pharmacy, Medical University of Gdańsk, 107 Hallera St., 80-416 Gdańsk, Poland; katarzyna.kimel@gumed.edu.pl (K.K.); barbara.sparzak-stefanowska@gumed.edu.pl (B.S.-S.); sylwia.godlewska@gumed.edu.pl (S.G.); 2 Department of Bromatology, Faculty of Pharmacy, Medical University of Gdańsk, 107 Hallera St., 80-416 Gdańsk, Poland; justyna.osko@gumed.edu.pl (J.O.); malgorzata.grembecka@gumed.edu.pl (M.G.)

**Keywords:** *Symphytum officinale* L., leaves, roots, polyphenols, HPLC, TLC, TLC-DB, antioxidant activity, chemometric analysis (cluster analysis—CA, factor analysis—FA)

## Abstract

*Symphytum officinale* L. (Boraginaceae) is a plant with proven anti-inflammatory and analgesic activity on the musculoskeletal system. The traditional use of comfrey primarily refers to its roots, although some literature also mentions the leaves as an alternative plant material. Comparing the therapeutic potential of both plant materials requires additional data on the chemical composition of *S. officinale* leaves and their biological properties. The aim of the study was to analyze polyphenols in comfrey leaves of different origins and to assess their antioxidant and anti-inflammatory potential against comfrey roots, also collected from different sources. Polyphenol profiles were recognized by 2D TLC and HPLC-DAD-ESI-MS methods, and quantitative analysis was performed by the HPLC-UV/Vis (high performance liquid chromatograph with-ultraviolet/visible detection) method. The antioxidant activity was assessed using DPPH (2,2-diphenyl-1-picrylhydrazyl), FRAP (ferric reducing antioxidant power), and ABTS (2,2′-azino-bis(3-ethylbenzothiazoline- 6-sulfonic acid) diammonium salt) assays, and for leaves also using the TLC-DB (thin layer chromatography-direct bioautography) technique with the DPPH radical. Chemometric analysis to assess the relationship between the antioxidant activity and the origin of comfrey plant raw materials was performed. Factor analysis (FA) confirmed that geographic origin and cultivation conditions influenced the antioxidant content of both plant raw materials. The study results indicate that comfrey leaves can substitute for comfrey roots containing not only caffeic acid derivatives but also flavonoids, and exhibiting stronger antioxidant activity.

## 1. Introduction

Comfrey (*Symphytum officinale* L., Boraginaceae) is a plant used in traditional medicine since ancient times. This species is mainly a source of roots, which are plant raw material with proven anti-inflammatory and analgesic effects on the musculoskeletal system [1,2]. Various types of products containing comfrey root extracts (ointments, creams or liniments) are recommended for use in fractures, dislocations, sprains, muscle and joint pain, and contusions [2,3].

Numerous studies indicate, that the biological activity of comfrey extracts is related to the presence of various bioactive constituents—mainly allantoin, mucopolysaccharides and polyphenolic compounds, with rosmarinic acid as the dominant compound [1]. Research on the phenolic acids in comfrey root over the years has also shown the presence of caffeic acid, p-hydroxybenzoic acid, chlorogenic acid, gallic acid, ellagic acid, 5-*O*-feruloylquinic acid and p-coumaric acid. In recent years, research on phenolic acids has been continued using the HPLC-DAD-QTOF-MS/MS method indicating the presence in comfrey root of a number of ester derivatives of caffeic acid, namely the isomers of salvianolic/isosalvianolic acids A, B and C, constituting trimers and tetramers of caffeic acid [4,5]. As a result of the isolation of the above compounds from the comfrey root by Trifan et al. [6], they were classified as caffeic acid-derived lignans, namely, rabdosiin, a tetramer of caffeic acid, and two trimers of caffeic acid—globoidnan A and B. The group of comfrey lignan compounds also includes two other lignans, namely comfrein A and ternifoliuslignan D [7]. The literature data indicate the significant antioxidant potential of comfrey root extracts, which depends mainly on the extraction method and the type of solvent used [8,9,10].

Previous indications for the use of comfrey root orally, e.g., for stomach ulcers or externally for difficult-to-heal wounds, are no longer relevant due to the information about the presence of toxic pyrrolizidine alkaloids in the whole plant [1,11,12]. Pyrrolizidine alkaloids are not evenly distributed throughout comfrey—the concentration of these compounds in the roots is approximately 100 times higher than in the aerial parts. Depending on the plant part, the pyrrolizidine alkaloid content can range from 0.04% (leaves) to 0.6% (roots). The most toxic alkaloids found in comfrey are considered to be lasiocarpine, echimidine, and symphytine [13]. Pyrrolizidine alkaloids are absorbed in the gastrointestinal tract and then activated in the liver to intermediate pyrrole metabolites, which form cellular adducts with proteins and DNA. This, in turn, leads to hepatocyte cell membrane damage, hemorrhagic necrosis, veno-occlusive liver damage, and ultimately, liver failure [1].

Scientific data on the toxicity of these compounds (hepatotoxicity, pneumotoxicity, cytotoxicity and genotoxicity) were the basis for limiting the use of comfrey root only externally and on intact skin, in short-term therapies [1,11]. According to some researchers, the aerial parts of comfrey may constitute an alternative plant material to roots, demonstrating similar biological activity [2]; however, there is a limited number of comparative studies of both plant materials in this regard [6].

Among the plant raw materials obtained from comfrey, only the root has a monograph in the European Pharmacopoeia [14]. Products containing comfrey root preparations are registered in some EU countries as traditional medicinal products. Leaves, that are not included in the EMA monographs, are ingredients of various product categories mainly in herbal stores. Among them, Traumaplant ointment is available on the market, containing 10% juice from fresh, above-ground parts of *Symphytum* × *uplandicum*, free from pyrrolizidine alkaloids [15,16,17].

Single studies have shown that the chemical profile of comfrey leaves is similar to that of the root, particularly in terms of the presence of allantoin and some phenolic compounds [18,19]. The methanolic extract of the leaves showed the presence of gallic acid [8], while the aqueous extract contained caffeic acid, rosmarinic acid and gallic acid [19]. Comfrey leaves, like the roots, are also characterized by the presence of pyrrolizidine alkaloids, although these are present at much lower concentrations [11]. The literature data on the concentrations of compounds belonging to the individual groups of secondary metabolites indicate significant differences between these two plant materials. Unfortunately, previous studies mainly focus on a single raw material [4,5,7,8,18,19] and the scope of comparative analyses is insufficient [6,11].

Most studies on the antioxidant activity of comfrey have focused primarily on the roots. On the other hand, the antioxidant potential of leaves and roots has been determined in different studies, often using different extraction methods and solvents [4,8,9,20]. Therefore, it is impossible to compare the antioxidant properties of comfrey leaves and roots.

Considering the above data, a comparative qualitative and quantitative study of the chemical composition of comfrey leaves and roots was conducted in terms of phenolic compounds (including phenolic acids, flavonoids, and lignans). The study included comfrey leaves obtained from herbal companies (3), while comfrey roots were obtained from both commercial sources (4) and botanical gardens (4).

Using the HPLC-DAD-ESI-MS method, profiles of biologically active compounds from the polyphenol group in comfrey roots and leaves were identified and their content was determined using the developed HPLC-UV/Vis method. Flavonoid complexes in various leaf extracts were compared using 2D TLC. Antioxidant activity was assessed using and spectrophotometric methods—DPPH, FRAP, and ABTS [21] and also TLC-DB (direct bioautography) with DPPH radical [21]. Additionally, the cyclooxygenase 1 and 2 inhibitory properties were determined for rosmarinic acid, the main compound found in the analyzed plant materials, and for selected root and leaf extracts characterized by varying contents of caffeic acid derivatives.

The results of the phytochemical analyses and antioxidant activity studies were subjected to chemometric analysis to explain the relationship between the chemical composition and antioxidant activity, as well as the origin of the plant material, including cultivation conditions.

Studies have shown that comfrey leaves are characterized by a significantly higher content of rosmarinic acid compared to the roots, as well as the additional presence of flavonoid glycosides, which may be directly related to their higher antioxidant activity. These data indicate that comfrey leaves can be considered as a potential alternative to comfrey roots due to their stronger antioxidant activity, which is part of their anti-inflammatory action, which includes inhibition of cyclooxygenase-2. Spearman’s rank correlation revealed a strong correlation between rosmarinic acid and the antioxidant activity of comfrey roots and leaves. Moreover, CA separated the analyzed plant material according to the type of plant raw material, its origin, including geographical origin, and cultivation conditions affecting the level of antioxidants.

## 2. Materials and Methods

### 2.1. Chemicals

Acetonitrile Lichrosolv^®^, methanol Lichrosolv^®^, chloroform, methyl ethyl keton and ethyl acetate were purchased from Merck (Darmstadt, Germany). Formic acid LC-MS LichroPur 97.5–98.5%, was purchased from Sigma-Aldrich (Steinheim, Germany). Ultra-pure water for LC-MS analysis was obtained using Direct-Q^®^ Water Purification System (Merck Millipore; Darmstadt, Germany). Standard compounds: caffeic acid, rosmarinic acid, lithospermic acid, quercetin 3-*O*-glucoside, kaempferol 3-*O*-glucoside, quercetin 3-*O*-galactoside, kaempferol 3-*O*-galactoside, quercetin 3-*O*-(6″-malonyl-glucoside) were purchased from Sigma (Sigma-Aldrich, Steinheim, Germany).

### 2.2. Plant Material

The leaves of *Symphytum officinale* L. were obtained from three Polish herbal companies—HL1 (Podlaskie Voivodeship), HL2 (Podlaskie Voivodeship, Białowieża forest) and HL3 (Masovian Voivodeship).

The roots of *Symphytum officinale* L. were obtained from four commercial sources: herbal companies (Poland)—HR1 (Łódzkie Voivodeship, Mokrsko), HR2 (Podlaskie Voivodeship, Koryciny), HR3 (Podlaskie Voivodeship) and HR4 (Podlaskie Voivodeship, Białowieża forest), and four botanical gardens (Poland)—GR1 (Lublin), GR2 (Zabrze), GR3 (Łódź), and GR4 (Gdańsk). In total, 11 independent samples were included in the experiments, with three subsamples per sample (*n* = 11 × 3).

### 2.3. Extraction of the Analyzed Plant Raw Materials

#### 2.3.1. Extraction of Comfrey Leaves

Extracts were prepared by two different methods:

Method I: dried comfrey leaves (5 g) were powdered and extracted in a Soxhlet apparatus, first with chloroform (100 mL) to remove ballast compounds, and then with methanol (100 mL). The extracts obtained were filtered and diluted with methanol to a volume of 50 mL. Samples were stored at 4 °C until chromatographic analysis.
Method II: dried comfrey leaves (20 g) were ground and macerated for 48 h with methanol (600 mL), and then the solvent was evaporated. The residue was dissolved in water (25 mL) and extracted with ethyl acetate (3 × 15 mL). The organic layer containing polyphenols was filtered and evaporated to dryness, and then dry residue was dissolved in 10 mL of methanol and stored at 4 °C until chromatographic analysis.

#### 2.3.2. Extraction of Comfrey Roots

Comfrey root extracts were prepared by extraction of powdered plant raw material (HR1, 1 g) with methanol (25 mL) under the reflux condenser (70 °C, 15 min) (method III). The extracts obtained were filtered and filled to 25 mL with methanol. The samples were kept at 4 °C until chromatographic analysis.

### 2.4. HPLC-DAD-ESI-MS Analysis of the Obtained Extracts from Comfrey Leaves and Roots

The HPLC system used was by Shimadzu (Kyoto, Japan) and consisted of two pumps LC-20AD, semi-micro mixer, CBM-20A system controller, CT0-20AC column thermostat, SIL 20AC_XR_ autosampler, UV/Vis detector (Diode Array Detector) SPD-M20A, and LCMS-2020 mass spectrometer (Shimadzu Corp., Kyoto, Japan) with ESI ionization. Data were acquired and processed by LabSolution software version 5.89 (Copyrights© 2008–2016 Shimadzu Corporation, Kyoto, Japan).

For optimization purposes, Kinetex C-18 column, Kinetex PFP and Kinetex F5 columns (100 mm × 4.6 mm, 2.6 µm) were tested (Phenomenex, Torrance, CA, USA). Chromatographic separation was performed on Kinetex C-18 column (the extracts from leaves) and Kinetex F5 column (the extracts from roots). The mobile phase consisted of water + 0.1% formic acid (A) and acetonitrile–water (50:50) + 0.1% formic acid (B). A linear gradient program at the flow rate of 0.8 mL/min was used for: 0 min–12% B, 10 min–20% B, 30 min–43% B, 50 min–100% B, from 60 min to 70 min–12% B. The injection volume was 1 µL, the column temperature was maintained at 35 °C and the separated compounds were recorded at the wavelength of λ—254 nm.

The method of collecting chromatographic data was SIM (single ion monitoring) and full scan in the range *m*/*z* 150–1000 in positive and negative ion mode. Mass detector operating parameters: ionization voltage: 4.5 kV (positive ionization) or 3.5 kV (negative ionization), detector voltage: 1.3 kV (+) or 3.5 kV (–), DL temperature 250 °C, heating block temperature 200 °C, spraying gas flow (N_2_) 1.5 L/min, drying gas flow (N_2_) 15 mL/min.

The individual compounds were identified by comparison of the UV spectra, mass spectra, and retention times to those of standard compounds (Table 1 and Table 2). Globoidnan A used as a reference compound was isolated from comfrey root (compound **5R**) (see below, Section 2.7).

### 2.5. HPLC-UV/Vis Quantitative Analysis of Phenolic Compounds in Extracts from Leaves and Roots of Comfrey

Quantification of phenolic acids (caffeic acid, rosmarinic acid) and flavonoids in *S. officinale* leaves and roots extracts was carried out by HPLC coupled with UV/Vis detector (LC-20AD with five-channel valve, DGU-20A5 degasser, CBM-20A system controller, CT0-10AS_VP_ column thermostat, SIL 20AC_XR_ autosampler, SPD-20A). Quantitative determination of analyzed compounds was based on their peak areas and comparison with a calibration curve for the corresponding standards at six concentrations—3.125–100 µg mL^−1^ for caffeic acid and rosmarinic acid; 1.560–100 µg mL^−1^ for quercetin 3-*O*-glucoside, kaempferol 3-*O*-glucoside and quercetin 3-*O*-galactoside. Triplicate injections were made for each sample. The results are expressed in mg or µg per g of dry weight (depending on the group of compounds) (Table 3 and Table 4). Regression equations were as follows—for caffeic acid—y = 1641.8x − 12,817; for rosmarinic acid—y = 3191.4x − 12,526 (also used for the quantitative determination of globoidnan A and B); for quercetin 3-*O*-glucoside—y = 1596x − 1198.1 (R^2^ = 0.9995); for kaempferol 3-*O*-glucoside—y = 1524.2x − 61.522 (R^2^ = 0.9998); for quercetin 3-*O*-galactoside (hyperoside)—y = 1969.5x − 2568.1 (R^2^ = 0.995) (Table 5).

#### Method Validation

The HPLC method developed for the purposes of quantitative analysis was validated for caffeic and rosmarinic acid, quercetin 3-*O*-glucoside and kaempferol 3-*O*-glucoside by determining calibration curves, limit of quantitation (LOQ) and limit of detection (LOD), according to ICH guidelines [22]. The precision of the HPLC method and recovery for rosmarinic acid were determined. Linearity for the working concentrations of the standard compounds was evaluated by determining the correlation coefficient. LOQ and LOD were determined as the concentration of the standard compound equaling 10× and 3× of the signal-to-noise ratio, respectively. The intra- and inter-day precision was evaluated by analyzing continuous injections of the same sample six times per day and once per day for six consecutive days, respectively, and determined according to the relative standard deviation (RSD). Recovery was evaluated by addition of known amounts of rosmarinic acid to analyze samples at three levels—50%, 100% and 150%. Validation parameters are presented in Table 5.

### 2.6. TLC Analysis of the Obtained Extracts from Comfrey Leaves and Roots

TLC analysis was carried out on TLC Si60F_254_ glass plates (10 × 10 cm), using the following mobile phases—chloroform–methanol–formic acid–water (70/30/2/2 *v*/*v*/*v*/*v*) (the first direction) and methyl ethyl ketone–ethyl acetate–formic acid–water (35/50/10/5 *v*/*v*/*v*/*v*) (the second direction). Samples were applied as spots in a volume of 15 μL using an AS-30 sample applicator (Desaga, Numbrecht, Germany). Chromatograms were developed using an automatic development chamber 2 (ADC2) (Camag, Muttenz, Switzerland) with saturation 10 min. The obtained chromatograms were observed under UV light at λ—366 nm after derivatization with a 1% solution of 2-aminoethyl diphenylborate (natural product reagent; NPR) in methanol and then with a 5% solution of Macrogol 400 (PEG) in ethanol [14], respectively, using derivatizer for automated reagent spraying (Camag, Muttenz, Switzerland).

#### TLC-Bioautography Assay with DPPH Reagent

Determinations of free radical scavenging properties using the DPPH^•^ radical were carried out according to the methodology described by Jesionek et al. [23]. Chromatograms developed on TLC Si60F_254_ glass plates were immersed in a solution of 0.05% DPPH in methanol and examined in daylight after 30 min of incubation in the dark. Free radical scavengers appeared as pale yellow spots on a purple background [24].

### 2.7. Isolation and Identification of Globoidnan A by a Use of Automated Solid-Phase Extraction (SPE)

**Figure 1:**
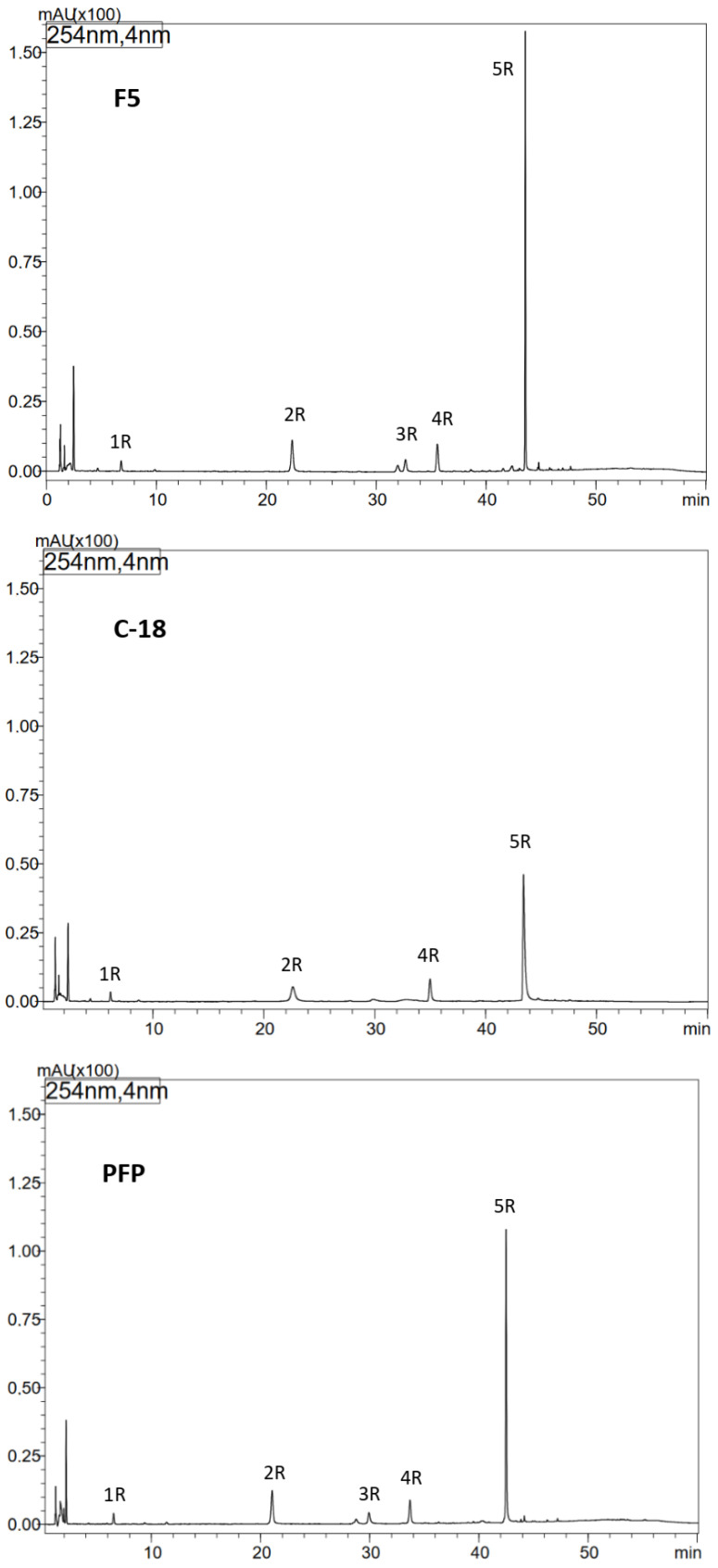
Optimization of HPLC separation of phenolic compounds from methanol extract of comfrey root (HR1) using different types of HPLC columns: Kinetex F5, Kinetex C-18 and Kinetex PFP columns, UV detection λ—254 nm. Compound numbers—according to Table 1.

A quantity of 3 g of dried comfrey roots (HR1) was extracted according to method III (Section 2.3.2). Next, the solvent from the obtained extract was evaporated under reduced pressure and the dry residue was dissolved in 24 mL of 30% methanol. Globoidnan A, previously described as a constituent of comfrey root [7,25], was separated as the compound named on HPLC chromatogram as **5R** (Figure 1) with the use of an automated solid-phase extraction (SPE) system (Horizon SmartPrep Extractor, Biotage^®^, Horizon Technology, Salem, NH, USA). The extract each time (n-15) was applied in the volume of 1 mL into DSC-18 column (1 g, 6 mL) previously conditioned with methanol, water and 30% methanol (6 mL of each), at the flow rate 1 mL/min and fractionated with the increasing concentrations of methanol: fractions I–III with 2 mL of 30% methanol each, fractions IV–VI with 2 mL of 50% methanol each and fraction VII with 6 mL of methanol. Fractions IV and V from the subsequent separations (n-15), containing globoidnan A, were controlled by the HPLC-UV/Vis method established by us (Section 2.4), then combined and lyophilized after the evaporation of methanol. The structure of compound **5R** as globoidnan A was confirmed by NMR and mass spectrometry data and the obtained data were consistent with the literature data [7,25,26].

### 2.8. Determination of Antioxidant Activity Using Spectrophotometric Methods

Antioxidant activity was determined using plant extracts prepared according to the procedures described above, appropriately diluted depending on the assay used, as follows:

• Comfrey roots—dilutions with methanol 1:10 (DPPH) and 1:20 (FRAP, ABTS)
• Comfrey leaves (method I)—dilution with methanol 1:50 (DPPH, FRAP, ABTS).

#### 2.8.1. Stable 2,2-Diphenyl-1-picrylhydrazyl (DPPH^•^) Radical Test

The assessment of antioxidant activity was performed using a 0.04 mM methanol solution of the stable 2,2-diphenyl-1-picrylhydrazyl radical (DPPH^•^) against a standard curve prepared from six dilutions of 6-hydroxy-2,5,7,8-tetramethylchroman-2 acid-carboxylic acid (Trolox) in methanol with concentrations: 0.02; 0.04; 0.05; 0.06; 0.08; 0.1 mM. In the given concentration range, the method was linear. The Trolox regression equations and R^2^ values were as follows: R^2^ = 0.99922; y = −3.0554x + 0.3776.

Before starting the measurements, the freshly prepared methanolic DPPH solution was incubated for 60 min at 4 °C. Then, 2.5 mL of DPPH solution and 350 μL of the given analyte were combined in quartz cuvettes (tested extract/dilution of Trolox − standard curve/methanol − blank sample), the prepared mixtures were incubated for 30 min in a dark place at room temperature, and then spectrophotometric measurements were made at wavelength λ—517 nm.

#### 2.8.2. Determination of the Ability to Reduce Iron (III) Ions—FRAP Test

The determination of the reducing capacity of iron (III) ions was carried out using the FRAP reagent, which consists of mixing in a ratio of 10:1:1 (*v*/*v*/*v*) and bringing to a temperature of 37 °C, as follows:

• Using a 300 mM acetate buffer solution, pH 3.6,
• A 10 mM solution of 2,4,6-tris(2-pyridyl)-1,3,5-triazine (TPTZ) in 40 mM hydrochloric acid (HCl),
• A 20 mM FeCl_3_ × 6H_2_O solution in water against a standard curve prepared from six dilutions of Trolox in water at the following concentrations: 0.02; 0.03; 0.06; 0.12; 0.36; 0.48 mM. In the given concentration range, the method was linear. The Trolox regression equations and R^2^ values: R^2^ = 0.99993; y = 2.0901x + 0.1568.

Before starting the measurements, the FRAP reagent was heated in a water bath to 37 °C. Then, 3 mL of FRAP reagent and 150 μL of the appropriate analyte were combined in quartz cuvettes (tested extract/dilution of Trolox − standard curve/water − blank sample), the prepared mixtures were incubated for 30 min in a dark place at room temperature and then a spectrophotometric measurement was performed at a wavelength λ—593 nm.

#### 2.8.3. Determination of Antioxidant Activity Using 2,2′-Azobis(3-ethylbenzothiazoline-6-sulfonate) Diammonium Salt—ABTS Test

In order to determine the antioxidant activity, the ABTS^•+^ cation radical was generated by incubating the mixture in a dark place at 4 °C for 15 h, as follows:

• A measured 2 mL of 7 mM solution of 2,2′-azobis(3-ethylbenzothiazoline-6-sulfonate) diammonium salt (ABTS),
• A measure of 0.35 mL of 140 mM potassium persulphate.

After the incubation time, the mixture was diluted with redistilled water in the ratio 1: 90 (*v*/*v*) while maintaining a constant absorbance of 0.7 ± 0.02. Measurements were carried out against a standard curve prepared from six dilutions of Trolox in water at the following concentrations: 0.02; 0.03; 0.05; 0.08; 0.1; 0.12 mM. In the given concentration range, the method was linear. The Trolox regression equations and R^2^ values: R^2^ = 0.99941; y = −2.4664x + 0.6456

Measured samples of 2 mL of ABTS reagent and 200 μL of the appropriate analyte (tested extract/Trolox dilution − standard curve/water − blank sample) were combined in quartz cuvettes, then the absorbance was measured twice at a wavelength of 734 nm, 6 min after the start of the reaction.

### 2.9. Assessment of Cyclooxygenase-1 and -2 Inhibition

For testing anti-inflammatory activity, extracts from leaves were prepared according to method I described above (Section 2.3.1).

The activity of rosmarinic acid was determined using a standard substance (rosmarinic acid ≥ 98%, from *Rosmarinus officinalis*; Sigma-Aldrich, Darmstadt, Germany) dissolved in methanol and diluted with methanol to obtain concentrations of 10, 100, 500 μM, respectively. To determine the activity of methanol extracts of comfrey root and leaves obtained according to the procedures described above, appropriate dilutions with methanol were prepared as follows:

• Comfrey roots—dilutions 1:10, 1:20, 1:50;
• Comfrey leaves—dilutions 1:10, 1:20, 1:50, 1:100, 1:200.

Cyclooxygenase-1 and -2 (COX-1, COX-2) inhibition was assessed using commercial COX inhibitor screening kits (ovine/human) (Cayman Chemical, Ann Arbor, MI, USA) according to the procedures described [27]. The absorbance of the test sample was measured after 20 or 30 min, with the value of the control sample ranging from 0.3 to 2.0. The measurement was performed at wavelength λ—405, λ—410, λ—415, and λ—420 nm.

### 2.10. Statistical Analysis

Statistical analyses were carried out using Statistica 13.3 software. Caffeic acid and rosmarinic acid levels, as well as the outcomes of the DPPH, FRAP, and ABTS procedures for the comfrey leaves and roots, were among the information used in the analysis. The results of compounds like globoidnan A and B, which are absent from the leaves but were also examined in the roots, were additionally taken for analysis. The data were classified into two main groups for statistical analysis. The first group consisted of the findings for the roots of comfrey, and the second group consisted of the outcomes for the roots and leaves. The data were analyzed for the existence of a normal distribution. In its absence, non-parametric tests such as the Kruskal–Wallis test and Spearman rank correlation were used. The resulting database was used to perform factor analysis (FA) and cluster analysis (CA) using Ward’s method and Euclidean distance. Samples of comfrey roots were differentiated in view of geographical origin and cultivation method (producer–garden). Leaves and roots, on the other hand, were discriminated by anatomical part (root–leaf), origin, and cultivation method for the content of the analyzed compounds. The number of samples analyzed in each group: roots (*n* = 8 × 3), root-leaf (*n* = 11 × 3), geographical origin (*n* = 11 × 3), producer vs. garden (*n* = 4 × 3 vs. 4 × 3, respectively).

## 3. Results

### 3.1. Optimization of Extraction Methods

In the initial stage of the research, the conditions for extracting active compounds from dried comfrey roots and dried leaves were optimized. Depending on the type of plant material analyzed, different extraction techniques were used, namely liquid–solid extraction under reflux and ultrasound-assisted extraction for comfrey roots, and Soxhlet extraction, ultrasound-assisted extraction and maceration for comfrey leaves. Alcohols and their mixtures with water were used as extraction solvents—methanol, ethanol, ethanol 60% and methanol 50%. Extraction of comfrey root using a mixture of water and alcohol resulted in a highly viscous extract due to its high polysaccharide content. This was a serious limitation, especially in HPLC analysis, so methanol was ultimately chosen as the extractant. When optimizing the method of extracting active compounds from comfrey leaves, characterized by a high chlorophyll content, the plant raw material was pre-purified with chloroform in a Soxhlet apparatus and then extracted with methanol. Finally, extracts from comfrey roots for qualitative and quantitative analysis and biological studies were obtained by the liquid–solid extraction method under reflux with methanol, while in the case of comfrey leaves two extraction methods were used—extraction in a Soxhlet apparatus for qualitative and quantitative analysis and biological studies (method I) and maceration for the qualitative analysis of phenolic compounds (method II).

### 3.2. HPLC-DAD-ESI-MS Analysis of Extracts from Comfrey Roots

To improve the efficiency of HPLC separation of phenolic compounds present in comfrey root extracts, tests were conducted using three types of HPLC columns: Kinetex C-18, PFP, and F5. The best resolution was obtained using the Kinetex F5 column (a larger number of well-resolved, narrow-base peaks were observed in the chromatograms, especially in the t_R_ range of 36–48 min) (Figure 1). Under the developed conditions, in relation to the reference compounds, the presence of lithospermic acid in comfrey root extracts was excluded, while the presence of caffeic acid (compound **1R**; Table 1) and rosmarinic acid (compound **4R**; Table 1) was confirmed. Based on the UV and ESI-MS spectra of compounds **2R** and **3R** obtained by HPLC-DAD-ESI/MS (Table 1), they were identified as caffeic acid trimer—globoidnan B ([M − H]^−^/[M + H]^+^ at *m*/*z* 537/539) and caffeic acid tetramer—rabdosiin ([M − H]^−^/[M + H]^+^ at *m*/*z* 717/719), respectively. The high peak intensity of compound **5R** (Figure 1) indicated its high content in the extract, therefore its isolation was performed from comfrey root extract HR1 using an automated solid-phase extraction (SPE) system. Chromatographic data obtained by HPLC-DAD-ESI/MS—UV and ESI-MS spectral data ([M − H]^−^/[M + H]^+^ at *m*/*z* 493/491) indicated that it is globoidnan A (Table 1), previously described by Trifan et al. and D’urso et al. [7,25].

### 3.3. Isolation of Unknown Compound ***5R*** from the Comfrey Root Extract (HR1) Using an Automated Solid Phase Extraction (SPE) System

Taking into account the t_R_ value of compound **5R**—approx. 44 min on the Kinetex C-18 column with the gradient elution program used (Figure 1)—it was calculated that its elution takes place at a concentration of approx. 35% acetonitrile in the mobile phase, which corresponds to the elution strength of 40% methanol (based on the differences in the elution strength of both solvents) [28]. The developed isolation program on the DSC-18 column included elution with 30% methanol, next with 50% methanol, and finally with 100% methanol. Compound **5R** was obtained in fractions IV-V of 50% methanol, and its structure as globoidnan A was confirmed based on 2D NMR spectra—COSY, ROESY, HSQC and HMBC, which were consistent with the literature data previously described for this compound [7,25].

### 3.4. HPLC-DAD-ESI-MS Analysis of Extracts from Comfrey Leaves

**Figure 2:**
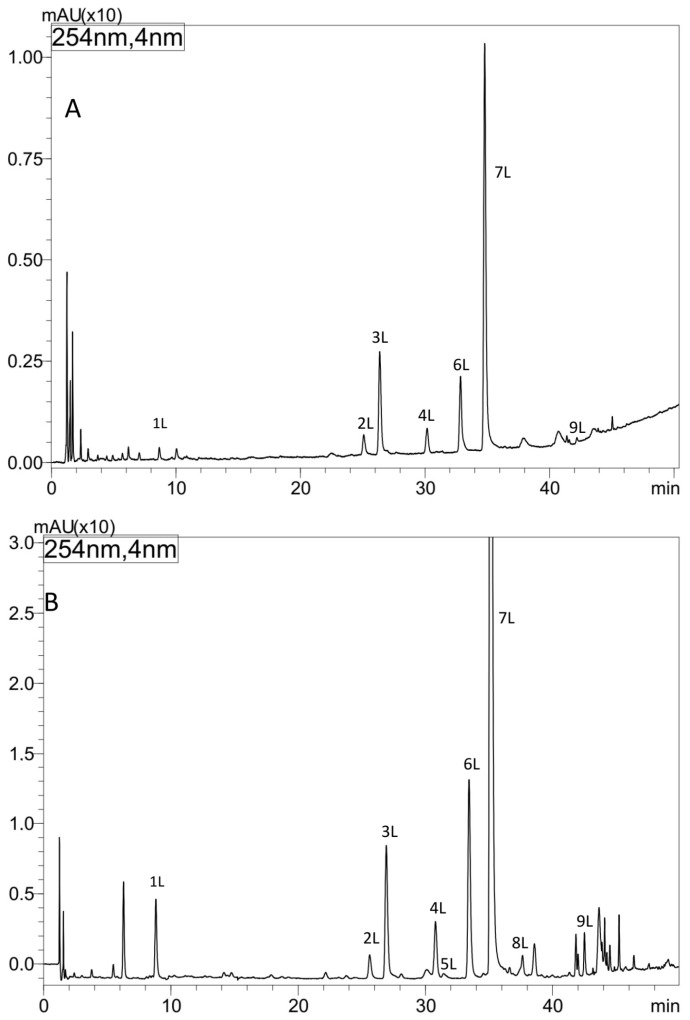
HPLC separation of extracts from comfrey leaves—HL3 (method I, II) prepared by (**A**) Soxhlet extraction with methanol after purification of plant raw material with chloroform (method I), (**B**) maceration with methanol followed by LLE with ethyl acetate (method II). Column: Kinetex C-18, detection: UV λ—254 nm.

**Figure 3:**
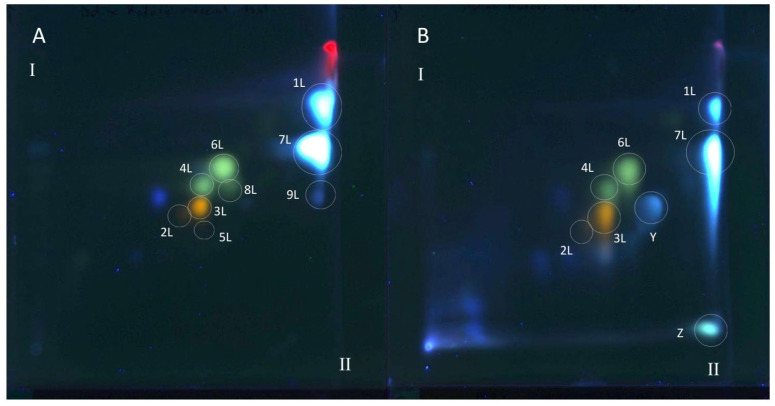
The 2D TLC separation of extracts from comfrey leaf HL obtained by (**A**) maceration with methanol followed by LLE with ethyl acetate (method II); (**B**) Soxhlet extraction with methanol after purification of plant raw material with chloroform (method I). Numbers of the compounds correspond to Table 2. TLC Si60_F254_ glass plates; mobile phases—chloroform–methanol–formic acid–water (70/30/2/2; *v*/*v*/*v*/*v*) (the first direction—I) and methyl ethyl ketone–ethyl acetate–formic acid–water (35/50/10/5; *v*/*v*/*v*/*v*) (the second direction—II), derivatized with NPR, UV—366 nm.

The metabolic profiles of comfrey leaf extracts prepared by two different methods were compared—Soxhlet extraction with methanol after purification of the plant material with chloroform (method I) and maceration with methanol followed by LLE with ethyl acetate (method II) (Figure 2). Chromatographic data of phenolic compounds separated and identified in both extracts, their retention times, absorption maxima in the UV spectra and *m*/*z* values of molecular ions in positive and negative mode, are summarized in Table 2. Nine compounds were identified (compounds **1L**–**9L**), including seven recognized with reference to the standards, namely caffeic acid (**1L**), rosmarinic acid (**7L**), quercetin 3-*O*-glucoside (**3L**), kaempferol 3-*O*-glucoside (**6L**) quercetin 3-*O*-galactoside (**2L**), kaempferol 3-*O*-galactoside (**4L**) and quercetin 3-*O*-(6″-malonyl-glucoside) (**5L**). Based on the elution order of quercetin and kaempferol derivatives (the presence of an additional hydroxyl group in the quercetin structure determines the shorter t_R_ value, in contrast to the t_R_ value of kaempferol derivatives), compound **8L**, with molecular ions [M − H]^−^ at *m*/*z* 533 and [M + H]^+^ at *m*/*z* 535 and UV spectra corresponding to kaempferol derivatives (λ_max_ 264, 294sh and 341 nm), was tentatively identified, next to quercetin 3-*O*-(6″-malonyl-glucoside) as another malonic acid ester, namely kaempferol 3-*O*-(6″-malonyl-glucoside). In the UV spectra of both malonic esters of quercetin and kaempferol, the shift of the absorption maximum I towards shorter wavelengths, which is characteristic for flavonol esters with phenolic acids, was not observed [29]. Two flavonol esters—quercetin 3-*O*-(6″-malonyl-glucoside) (**5L**) and kaempferol 3-*O*-(6″-malonyl-glucoside) (**8L**) were detected only in the extract obtained by maceration with methanol (method II) (Figure 2 and Figure 3).

Based on the obtained UV spectral data, *m*/*z* value of molecular ion [M − H]^−^ at *m*/*z* 491 the compound **9L**, was identified as globoidnan A—a trimer of caffeic acid, previously described in comfrey root [7,25]. Its identity was confirmed by comparison with globoidnan A, isolated by us from comfrey root (method described in Section 2.7).

**Figure 4:**
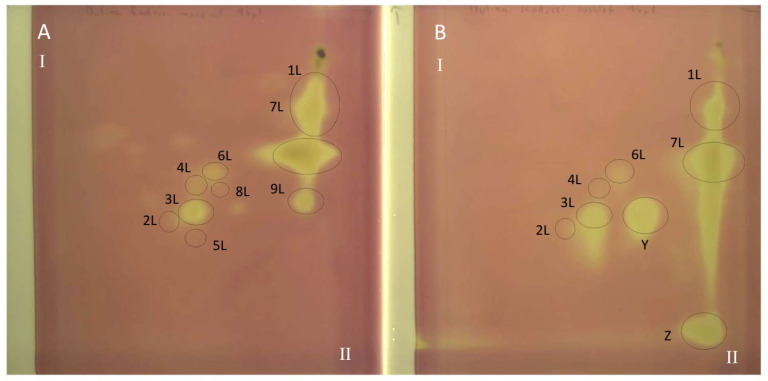
The 2D TLC-DB with DPPH radical chromatogram of extracts from comfrey leaf HL obtained by (**A**) maceration with methanol followed by LLE with ethyl acetate (method II); (**B**) Soxhlet extraction with methanol after purification of plant raw material with chloroform (method I). TLC Si60_F254_ glass plates; mobile phases—chloroform–methanol–formic acid–water (70/30/2/2; *v*/*v*/*v*/*v*) (the first direction—I) and methyl ethyl ketone–ethyl acetate–formic acid–water (35/50/10/5; *v*/*v*/*v*/*v*) (the second direction—II). Yellow spots represent compounds with free radical scavenging activity, visible light. Numbers of the compounds correspond to Table 2.

**Figure 5:**
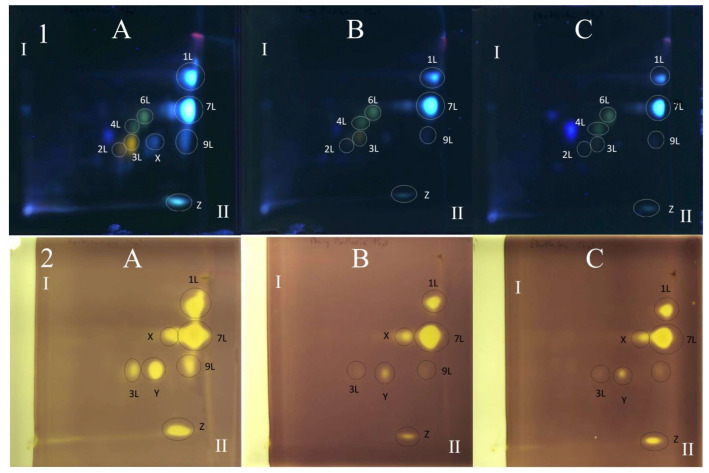
The 2D TLC separation of comfrey leaf extracts of different origins (HL1–3) (15 μL) prepared by Soxhlet extraction (method I): TLC Si60_F254_ glass plates; mobile phases—chloroform–methanol–formic acid–water (70/30/2/2; *v*/*v*/*v*/*v*) (the first direction—I) and methyl ethyl ketone–ethyl acetate–formic acid–water (35/50/10/5; *v*/*v*/*v*/*v*) (the second direction—II), derivatized with NPR, UV 366 nm. (**1**) Derivatized with NPR, UV λ—366 nm: (**2**) Derivatized with DPPH reagent—yellow spots represent compounds with free radical scavenging activity, visible light. Numbers of the compounds correspond to Table 2. (**A**) HL3, (**B**) HL1, (**C**) HL2.

All polyphenolic compounds (**1L**–**9L**) identified by the HPLC-DAD-ESI-MS method were observed on TLC chromatograms (Figure 3, Figure 4 and Figure 5) and numbered according to the peak numbers in the HPLC chromatogram (Table 2, Figure 2). However, it is worth noting that other unidentified compounds **X**, **Y**, **Z** with light blue and blue fluorescence under UV light at 366 nm after spraying with the natural product reagent were also visible in the 2D TLC chromatograms, especially of the extract obtained by extraction in Soxhlet apparatus (method I) (Figure 5).

### 3.5. Quantitative Analysis of Biologically Active Compounds in Extracts from Comfrey Roots and Leaves by HPLC-UV/Vis

#### 3.5.1. Comfrey Root

Quantitative analysis of caffeic acid and its three derivatives—globoidnans A and B and rosmarinic acid was performed in extracts from roots originated from commercial sources (HR 1–4) and botanical gardens (GR 1–4) (Table 3 and Table 4).

The developed method was validated in terms of linearity, limits of detection (LOD) and quantification (LOQ), one-day and inter-day precision, showing that the method is precise and linear and is characterized by satisfactory recovery (Table 5). The obtained results indicate significant differences in the content of caffeic acid derivative esters in the analyzed plant raw materials from different sources (Table 3).

Caffeic acid was present in the lowest concentrations in comfrey roots—the average concentration of this compound was 0.089–0.11 mg/g d.w. Plant raw materials from botanical gardens (GR1, GR3, GR4) were characterized by a statistically significant lower content of caffeic acid (0.09–0.10 mg/g d.w.), compared to plant raw materials obtained from herbal companies (HR 1–4—0.11 mg/g d.w.) (Table 3). The exception were GR2 roots from the botanical garden, with a caffeic acid content at the level of commercial plant raw materials. This relationship was not observed in the case of the remaining compounds determined—esters of caffeic acid derivatives.

The content of rosmarinic acid ranged from 0.49 to 1.80 mg/g d.w. However, in the two plant raw materials HR3 and GR4, rosmarinic acid was not the dominant compound, occurring in lower concentrations than globoidnan A (HR3) or globoidnan B (GR4). The content of globoidnan A ranged from 0.20 to 1.08 mg/g d.w., while that of globoidnan B from 0.34 to 0.92 mg/g d.w. (Table 3).

#### 3.5.2. Comfrey Leaves

All analyzed comfrey leaf samples, compared to the roots, were characterized by significantly higher contents of caffeic acid (0.139–0.194 mg/g d.w.) and rosmarinic acid (1.91–2.41 mg/g d.w.) (Table 3 and Table 4). Additionally, the content of four flavonoid glycosides, i.e., quercetin and kaempferol-3-*O*-glucosides and 3-*O*-galactosides, was determined (Table 4), with significant differences in their contents observed. HL3 leaves were characterized by more than twice as high content of quercetin-3-*O*-galactoside, kaempferol-3-*O*-galactoside, and kaempferol-3-*O*-glucoside, and almost four times as high concentration of quercetin-3-*O*-glucoside (Table 4). The content of flavonol glycoside esters and globoidnan A in the tested plant material was not determined due to the fact that they were identified only in the ethyl acetate fraction of the methanol extract HL3.

### 3.6. TLC-DB with DPPH Radical for Determination of Antioxidant Activity of Extracts from Comfrey Leaves

The TLC-bioautography with DPPH radical of comfrey leaf extracts prepared by two different methods: method I (HL 1–3) and II (HL-3), showed free radical scavenging activity of all identified compounds (Figure 4 and Figure 5). The strongest activity was shown by caffeic acid (compound **1L**) and rosmarinic acid (compound **2L**), which, based on their TLC spot intensity. These compounds are the dominant in both extracts. From the group of flavonoids derivatives, the strongest antioxidant activity was demonstrated by quercetin 3-*O*-glucoside (compound **3L**) and kaempferol 3-*O*-glucoside (compound **6L**), while the galactosides (compound **2L** and **4L**) and glucoside-malonylesters (compounds **5L** and **8L**, present only in extracts obtained with maceration) gave barely visible pale yellow spots as a result of DPPH radical scavenging.

Globoidnan A (compound **9L**), despite the low spot intensity after using the NPR reagent, in the TLC chromatogram, showed high DPPH radical scavenging activity in the bioautography test, equal to the spot intensity of quercetin 3-*O*-glucoside, a compound present in leaves in relatively higher concentrations (Figure 5). In addition to the identified compounds **1L**–**9L**, three unidentified compounds (compounds X, **Y**, and **Z**), visible as intense yellow spots on TLC chromatograms, compared to the other compounds demonstrated strong antioxidant activity (Figure 5). These compounds were present only in extracts prepared by Soxhlet extraction, and the intensity of their spots depended on the concentrations in the extract.

**Figure 6:**
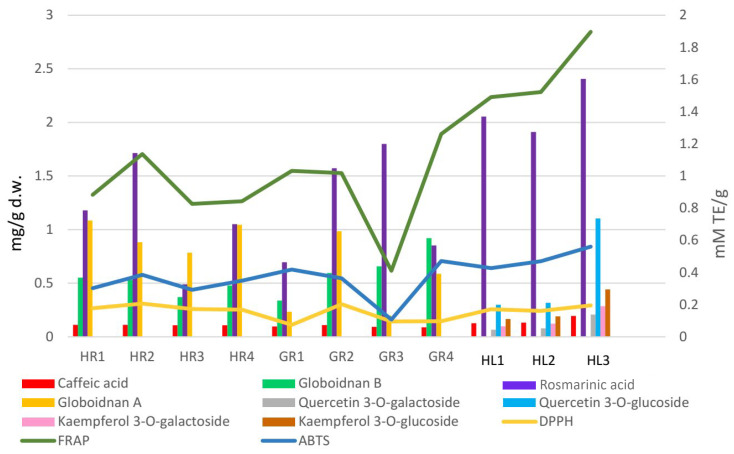
Interrelationship between the antioxidant activity [mmol TE/g] of the comfrey plant raw materials and the content of phenolic compounds [mg/g d.w.].

Comfrey leaf extracts from different producers were characterized by differences in antioxidant activity determined for individual compounds separated on TLC chromatograms after spraying with DPPH solution (Figure 5). Comfrey leaf extract HL3 was distinguished by the strong intensity of yellow spots visible on the TLC chromatogram after spraying with DPPH solution, which also correlated with the highest content of caffeic acid derivatives and flavonoid compounds determined by HPLC (Table 4) and highest antioxidant activity (FRAP and ABTS assays) (Table 6, Figure 6).

### 3.7. Determination of Antioxidant Activity Using UV/Vis Spectroscopy Techniques

The analysis of the results of the antioxidant activity tests of methanol extracts from comfrey leaves and roots (Section 2.8) showed statistically significant differences between them (Table 6).

Methanol extracts of comfrey leaves were characterized by similar antioxidant activity in all three tests—DPPH, ABTS and FRAP. The FRAP test showed that they were characterized by a 2–4 times higher capacity to reduce iron ions compared to methanol extracts from comfrey roots (1.491–1.897 mmol TE/g d.w. vs. 0.412–1.26 mmol TE/g d.w.)—statistically significant differences were revealed between most of the analyzed comfrey root extracts, with the exception of the extract HL1. At the same time, in DPPH and ABTS tests, comfrey root extracts showed similar free radical scavenging activity as extracts from underground parts of comfrey. Antioxidant activity measured by the DPPH test did not show any statistically significant differences between comfrey leaves and roots obtained from herbal companies (HR1–4). In turn, the antioxidant activity of leaf extracts measured by the ABTS test was similar to that of root extracts from botanical gardens (GR1, GR2, GR4) (Table 6, Figure 6).

Greater variability in antioxidant potential was noted for comfrey roots. Roots from domestic herbal producers had significantly higher antioxidant activity in the DPPH test (almost twice as high) than roots collected in botanical gardens, with the exception of GR3 roots.

The GR1 root extract showed the weakest activity in the FRAP and ABTS tests and, alongside GR2 and GR4 extracts, the weakest antioxidant activity in the DPPH test. It is worth noting that, among the roots tested, it had the lowest content of globoidnan B and one of the lowest contents of globoidnan A and rosmarinic acid (Table 2). Similarities in the strength of antioxidant activity were noted for methanol extracts of comfrey roots from botanical gardens (GR2, GR3, GR4) (Table 6, Figure 6).

#### 3.7.1. Correlations

Comfrey leaves were characterized by significantly higher antioxidant activity than comfrey roots, especially in terms of the ferric reducing activity measured by the FRAP assay. Spearman’s rank correlation test was performed at three levels of significance: *p* < 0.05, *p* < 0.01 and *p* < 0.001. For descriptive purposes, the strength of selected associations is additionally expressed as R^2^ values, calculated as the square of Spearman’s correlation coefficient (ρ^2^), without implying any causal relationship. In comfrey root samples, the strongest statistically significant correlations (*p* < 0.001) were observed for the following variable pairs: caffeic acid–globoidnan A (R^2^ = 0.684), rosmarinic acid–FRAP (R^2^ = 0.660), and ABTS–FRAP (R^2^ = 0.879). Considering both root and leaf samples, significant correlations (*p* < 0.001) were observed between caffeic acid and rosmarinic acid (R^2^ = 0.688), caffeic acid and FRAP (R^2^ = 0.674), rosmarinic acid and FRAP (R^2^ = 0.864), as well as between rosmarinic acid and ABTS (R^2^ = 0.739). In addition, a strong correlation was found between FRAP and ABTS (R^2^ = 0.910), which is consistent with the fact that both assays evaluate antioxidant capacity based on similar electron-transfer mechanisms.

#### 3.7.2. Kruskal–Wallis Test

The Kruskal–Wallis non-parametric test showed statistically significant differences in the two groups of data analyzed (roots and root–leaf) in terms of geographical origin, cultivation method, and differentiation between anatomical parts of the comfrey plant.

The results obtained for the group of roots in view of geographical origin were as follows: caffeic acid (H = 17.520; *p* = 0.014), globoidnan B (H = 20.413; *p* = 0.005), rosmarinic acid (H = 21.773; *p* = 0.003), globoidnan A (H = 19.693; *p* = 0.006), DPPH (H = 20.004; *p* = 0.006), FRAP (H = 19.933; *p* = 0.006), and ABTS (H = 19.880; *p* = 0.006). The root data classified according to the cultivation method (producer–garden) were as follows: caffeic acid (H = 9.363; *p* = 0.002), globoidnan B (H = 3.203; *p* = 0.073), rosmarinic acid (H = 0.403; *p* = 0.525), globoidnan A (H = 6.453; *p* = 0.011), DPPH (H = 5.470; *p* = 0.019), FRAP (H = 1.080; *p* = 0.030), and ABTS (H = 1.470; *p* = 0.225).

The second group concerned root and leaf data, which were analyzed for the anatomical part of the plant, geographical origin, and cultivation method. The results of the Kruskal–Wallis root–leaf test (the anatomical part of the plant) were as follows: caffeic acid (H = 19.059; *p* = 0.000), rosmarinic acid (H = 19.059; *p* = 0.000), DPPH (H = 1.105; *p* = 0.293), FRAP (H = 19.059; *p* = 0.000), and ABTS (H = 11.805; *p* = 0.001). For the same group of data, the test was performed by taking into account another grouping variable, i.e., geographical origin: caffeic acid (H = 24.383; *p* = 0.002), rosmarinic acid (H = 16.200; *p* = 0.040), DPPH (H = 21.567; *p* = 0.006), FRAP (H = 17.330; *p* = 0.027), and ABTS (H = 22.652; *p* = 0.004). The last analysis concerned the influence of the cultivation method (producer–garden) on the data, and the results were as follows: caffeic acid (H = 16.035; *p* = 0.000), rosmarinic acid (H = 2.590; *p* = 0.108), DPPH (H = 5.385; *p* = 0.020), FRAP (H = 1.815; *p* = 0.178), and ABTS (H = 0.169; *p* = 0.681).

#### 3.7.3. Post Hoc Dunn’s Test

Dunn’s test was applied as a post hoc procedure for multiple pairwise comparisons between groups, using three significance levels: *p* < 0.05, *p* < 0.01, and *p* < 0.001. As in the Kruskal–Wallis test, the data were classified into two groups: roots and root–leaf samples. Using the same scheme, the data were analyzed with respect to geographical origin, cultivation method (producer vs. garden), and anatomical part of the plant (for the root–leaf group).

For the first set of comfrey root samples, statistically significant differences related to geographical origin were observed between samples from Zabrze and Lublin, with *p* = 0.0056 for DPPH and *p* = 0.0057 for FRAP. For data classified according to cultivation method, significant differences were observed for caffeic acid content between producer and garden samples (*p* = 0.0022).

In the root–leaf dataset, significant differences between roots and leaves were observed for caffeic acid (*p* = 0.0020), rosmarinic acid (*p* = 0.0040), FRAP (*p* = 0.0027), and ABTS (*p* = 0.0038). With respect to geographical origin, significant differences were found at the *p* < 0.01 level between samples from the Mazovian region (Kurpie) and Lublin for FRAP and ABTS (*p* = 0.0027 and *p* = 0.0038, respectively). The applied post hoc test for cultivation method (producer–garden) showed significant differences in caffeic acid content (*p* = 0.0001).

#### 3.7.4. Factor Analysis

**Figure 7:**
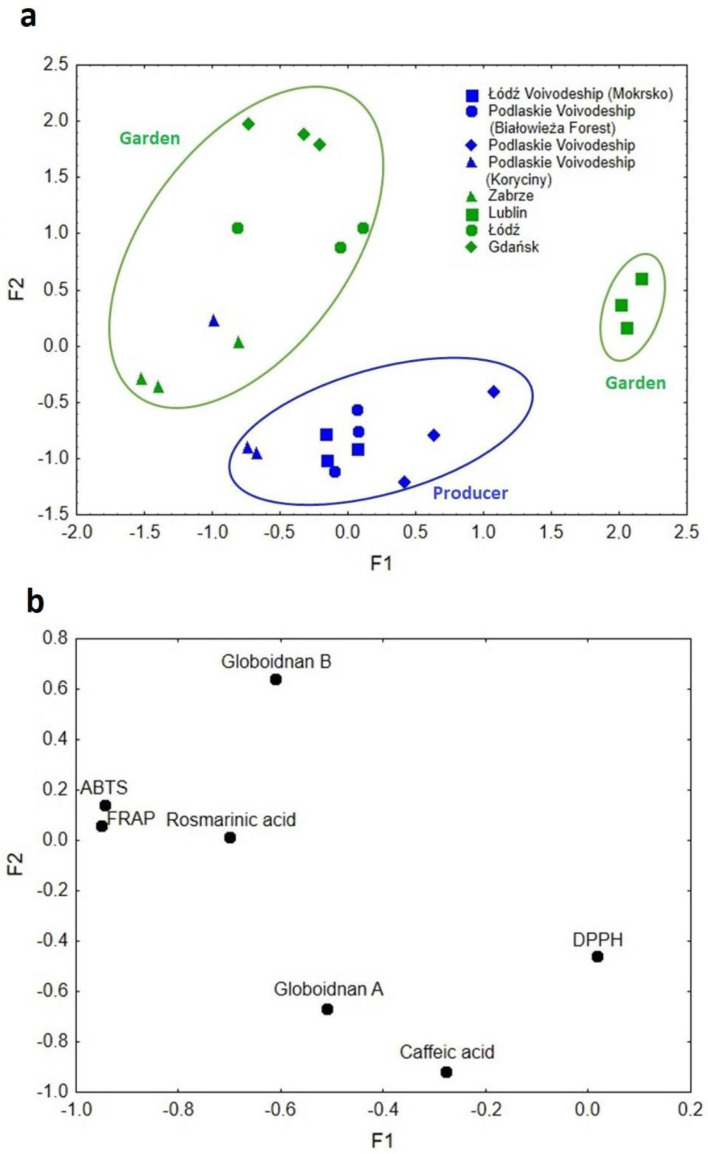
(**a**) Scatterplot of object samples of two factors in view of comfrey root geographical origin and cultivation method. (**b**) Scatterplot of loadings for elements in all the analyzed samples.

Factor analysis (FA) was performed for the data on comfrey roots, and its results are presented in Figure 7a,b. The FA resulted in factor one (F1) accounting for 42.80% of the explained variance and factor two (F2) accounting for 27.69%. The cumulative eigenvalue of the explained variance for both factors amounted to 70.49%. The resulting eigenvalues for F1 and F2 were 2.99 and 1.94, respectively. In Figure 7a,b, there was a clear diversification of the comfrey samples into three main groupings.

The first factor (F1) was responsible for the differentiation of samples in terms of their geographic origin. Samples originating from Lublin (south-east Poland) were clearly separated from the others and described with high F1 values. The DPPH method, which is characterized by greater sensitivity to hydrophobic compounds, was responsible for this differentiation [30].

The second grouping was described by low and medium F1 values, which corresponded to samples from Zabrze, Łódź and Gdańsk. Their locations on the map of Poland are arranged in a line, stretching from the north through the center to the south of the country, in general the western part of the country. These samples were characterized by such compounds as rosmarinic acid, globoidnan B, and the results of ABTS and FRAP methods (Figure 7b).

The medium values of F1 corresponded to samples from Podlaskie Voivodeship, which is situated in north-east Poland, described by globoidnan A and caffeic acid. The second factor (F2), on the other hand, was mainly responsible for the separation of the analyzed samples due to the method/conditions of plant cultivation (producer–garden). Low F2 values characterized samples from producers, mostly from Podlaskie Voivodeship, which were differentiated mainly by caffeic acid and globoidnan A. High F2 values were attributed to rosmarinic acid, globoidnan B, ABTS, and FRAP, and samples from plants grown under garden conditions. Such variations in soil and climatic conditions, as well as the plant’s harvest time, might have a likely impact [30]. The factor analysis carried out allowed the separation of the analyzed samples of comfrey roots in terms of geographic origin and the cultivation conditions used (producer–garden), affecting the proportion of individual antioxidants.

#### 3.7.5. Cluster Analysis

**Figure 8:**
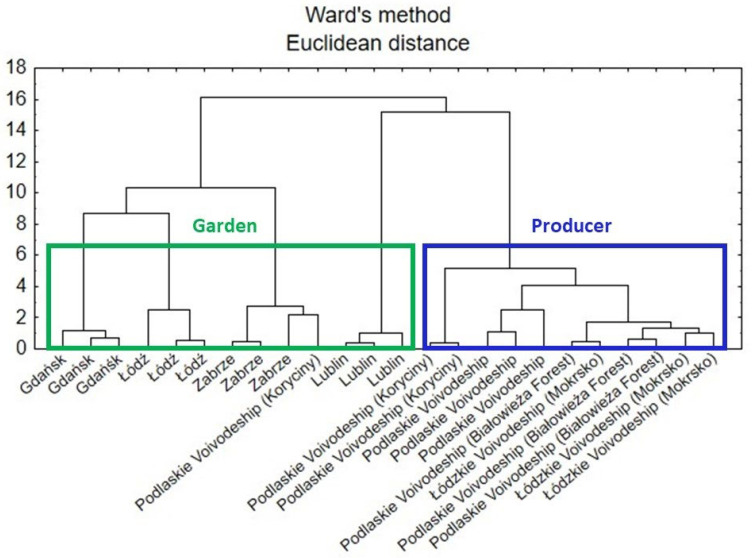
Hierarchical dendrogram for the comfrey root samples.

According to a dendrogram created by the cluster analysis (CA) (Figure 8), there are similarities between the comfrey samples (roots) in terms of how they were grown. Three main clusters can be distinguished. The two from the left (green line) concerned samples from gardens, whereas the one on the right (blue line) concerned samples obtained from various producers.

**Figure 9:**
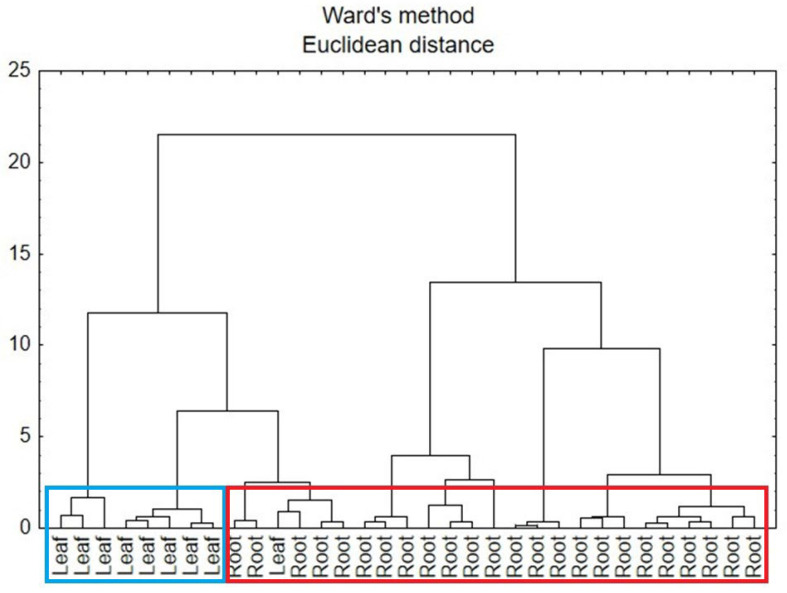
Hierarchical dendrogram for the root and leaf comfrey samples.

A second CA analysis was carried out based on the data for the roots and leaves of comfrey in terms of anatomical parts of the plant. In Figure 9, there can be identified two main clusters that represent the similarity of the samples to each anatomical part of the plant, i.e., leaf and root. However, part of the root samples was attributed to leaf cluster, which might be due to other factors influencing the composition of the plant.

### 3.8. Results of the Determination of the Anti-Inflammatory Activity of Rosmarinic Acid and Selected Extracts of Comfrey Roots and Leaves as Cyclooxygenase-1 and -2 Inhibitory Activity

The cyclooxygenase-1 and -2 inhibitory activity was assessed for rosmarinic acid, present in high concentrations in both comfrey root and leaf extracts. At the same time, IC_50_ (half maximal inhibitory concentration) values were determined, which were 300.36 μM for COX-1 and 1040.52 μM for COX-2, and indicated a stronger inhibitory activity against COX-1 (Table 7).

To determine the cyclooxygenase-1 and -2 inhibitory activity, a methanol extract from comfrey leaves HL3 was selected, which contained the highest concentrations of rosmarinic acid and flavonoids (Table 4). Among the methanol extracts from comfrey roots, the study included extracts from two plant raw materials that stood out from the rest, namely the HR3 extract with globoidnan A dominating the complex and the HR2 extract with the highest content of rosmarinic acid and a comparable content of globoidnan A (Table 3). Both methanol extracts from comfrey roots included in the study, similarly to rosmarinic acid, were characterized by higher cyclooxygenase-1 inhibitory activity compared to cyclooxygenase-2 and were observed for the HR2 extract 65.25% inhibition of COX-1 and 19.63% COX-2 inhibition at a concentration of 4 mg d.w./mL and for HR3 extract 55.7% COX-1 inhibition at a concentration of 4 mg d.w./mL and 28.86% COX-2 inhibition at a concentration of 8 mg d.w./mL (Table 8). An inverse relationship was observed for the methanol extract of comfrey leaves HL3 prepared by the Soxhlet extraction method (method I); namely, that the extract showed 70.93% inhibition of COX-2 at a concentration of 2 mg d.w./mL and 33.62% inhibition of COX-1 at a concentration of 10 mg d.w./mL (Table 8).

## 4. Discussion

Despite the long tradition of using comfrey root in medicine, there has been no systematic research on its chemical composition, especially in terms of phenolic and polyphenolic compounds. Only in recent years, studies conducted by D’urso et al. and Trifan et al. have provided new data on previously unknown compounds in this species, with the structure of caffeic acid trimers and tetramers [7,25]. The phytochemical analyses carried out as part of the presented research allowed for a more comprehensive understanding of the chemical profile of another plant raw material obtained from comfrey, i.e., its leaves. Our research used plant raw material from both national botanical gardens and plant raw materials from commercial sources available on the domestic herbal market. Phytochemical studies, based on chromatographic methods (HPLC, TLC), included qualitative and quantitative analysis of caffeic acid esters, which, among other compounds, contribute to the therapeutic effects of both analyzed plant raw materials—comfrey roots and leaves. In addition, the antioxidant activity of extracts from roots and leaves was determined and compared. The antioxidant activity of plant raw materials is part of their anti-inflammatory effect, because the development of inflammation is characterized by an increase in the level of free radicals. It was particularly interesting due to the presence of esters of caffeic acid derivatives, namely rhabdosin, globoidnan A and globoidnan B, which have previously been proven to possess strong antioxidant activity [7,25,31]. Furthermore, it has been shown in silico, that globoidnan A and globoidnan B may be COX-2 inhibitors [32].

The use of spectrophotometric tests—DPPH, ABTS and FRAP—allowed for the assessment of the total antioxidant potential of the tested plant extracts; therefore, additionally, the identification of individual compounds with antioxidant activity was carried out using the TLC-bioautography using the DPPH radical (Table 6, Figure 4, Figure 5 and Figure 6). The TLC-direct bioautography technique (TLC-DB) is a fast and cheap method for studying a wide range of biological properties of chemical components in plant extracts [33,34]. The DPPH test was used to assess the antioxidant activity of individual compounds in terms of their ability to scavenge free radicals [24,35]. Antioxidant activity is one of the biological activities exhibited by plant materials and is associated with the presence of phenolic compounds with redox potential, such as phenolic acids, flavonoids, xanthones, and lignans, which can act as reducing agents, hydrogen donors, metal chelators, or singlet oxygen quenchers. The mechanisms of antioxidant activity of compounds belonging to specific groups vary and are related to their chemical structure [36].

Phenolic acids, especially those derived from caffeic acid (rosmarinic acid, chlorogenic acid), and flavonoids, are groups of compounds with strong antioxidant activity, associated primarily with the presence of free -OH groups, capable of capturing and inhibiting the production of free radicals and reactive oxygen species (ROS). As a result, phenolic compounds protect cells against the harmful effect of oxidative stress, which participates in the development of inflammation. Phenolic acids additionally have anti-inflammatory activity by modulating the expression of pro-inflammatory cytokines and inhibitory effects on some enzymes, including cyclooxygenase-1 and -2 and/or lipoxygenase-5. The presence of phenolic acids in root and leaf extracts from comfrey can therefore determine their antioxidant and anti-inflammatory effects, and, as a consequence, anti-rheumatic and analgesic effects in the case of various types of musculoskeletal injuries (bruises, swelling) [1,9,20,36].

The literature data indicate a high antioxidant potential of comfrey root extracts, which has been shown to depend on the type of solvent used and the extraction method. A relationship has been demonstrated between the strength of the antioxidant effect and the content of polyphenols—it has been shown that polar extracts containing a larger amount of polyphenols have a stronger antioxidant effect [8,9,20]. In the case of comfrey root, antioxidant activity may be a significant component of its anti-inflammatory and wound-healing effects, directly impacting its medicinal value. Therefore, it is worthwhile determining the antioxidant potential of comfrey extracts and assessing the profile of bioactive compounds that may contribute to this effect.

Rosmarinic acid, alongside allantoin, is one of the most important biologically active constituents in comfrey roots and leaves. As an ester of caffeic acid and 3,4-dihydroxyphenyllactic acid, it exhibits antioxidant properties and inhibits the synthesis of prostaglandins and other proinflammatory proteins. Its antioxidant potential is related to its structure, which consists of two catechol groups (ortho-hydroxyl groups on two phenolic rings), an unsaturated bond, and a carboxylic acid, which allows it to donate electrons/protons to neutralize free radicals (ROS). The presence of two catechol structures (as in caffeic acid) is crucial because they provide readily donated hydrogen atoms and electrons that neutralize free radicals. The structure of rosmarinic acid contains several hydroxyl groups on aromatic rings, which are key for its antioxidant properties. An α,β-unsaturated carbonyl group system and a carboxylic acid group connect the two phenolic rings, contributing to its overall reactivity. Its electron-rich phenolic core makes it extremely effective at scavenging radicals, chelating metals, and enhancing the body’s internal antioxidant enzymes (such as superoxide dismutase—SOD, catalase—CAT) to combat oxidative stress and inflammation, making it valuable in health and food science [37,38].

Rosmarinic acid has been shown to have a protective effect on the skin, preventing UVB radiation-induced keratinocyte damage. Furthermore, data on the antifibrotic and hepatoprotective effects of rosmarinic acid are available in the literature, which, in the case of the therapeutic use of comfrey roots and leaves, may be fundamental in reducing the risk of adverse effects associated with the presence of hepatotoxic pyrrolizidine alkaloids with a 1,2-unsaturated necine skeleton [39,40,41,42]. Rosmarinic acid and its derivatives possess promising biological activities, such as improvement in cognitive performance, prevention of the development of Alzheimer’s disease, cardioprotective effects, reduction in the severity of kidney diseases and cancer chemoprevention [40]. Spearman’s rank correlation revealed a strong correlation between rosmarinic acid and the antioxidant activity of comfrey roots and leaves, revealed by the DPPH, ABTS and FRAP tests.

The results of HPLC analysis performed on comfrey roots confirmed the presence of rosmarinic and caffeic acids and the lignan trimer of caffeic acid—globoidnan A, the presence of which was previously described by D’urso et al. and Triffann et al. [7,25]. The developed isolation method using an automated SPE system is an alternative to the method described by Trifan et al., i.e., countercurrent chromatography (CCC) using a two-phase solvent system [25]. The conducted studies did not reveal the presence of lithospermic acid in the tested roots.

The literature data indicate that the content of rosmarinic acid in comfrey root ranges from 0.85 to 1.27 mg/g in aqueous extracts of this plant material to 1.94 mg/g in the water–ethanol extract [19,20,43,44]. The determined contents of rosmarinic acid in most methanol extracts are within this range (except for the plant raw materials HR3—0.49 ± 0.14 mg/g d.w. and GR1—0.70 ± 0.0227 mg/g d.w.). However, in two plant raw materials, rosmarinic acid was not the dominant compound, occurring at a lower concentration than globoidnan A (HR3) or globoidnan B (GR4) (Table 3). A high proportion of globoidnan A in phenolic complexes from comfrey root was also noticed by Trifan et al. [44], and it was the dominant compound in 6 of 16 analyzed hydroalcoholic extracts. However, the dominance of globoidnan B has not been reported so far.

Trifan et al. [45] showed that the storage time of the dried plant raw material is one of the potential reasons for the observed differences in the content of caffeic acid derivatives between comfrey roots. They found a decrease in the concentrations of individual compounds between 1 and 6 months after harvest—in the range of 30–47% for rosmarinic acid, 19–60% for globoidnan A, 25–50% for globoidnan B, and 29–63% for rabdosiin. However, the highest concentrations of some caffeic acid derivatives recorded in the second and third month of storage seem to contradict this thesis [45]. Our results do not take into account the effect of storage time on the content of caffeic acid derivatives, because the tested plant raw materials from botanical gardens were collected in a similar period (23 October–23 November 2018).

There are few studies on the chemical composition of the *S. officinale* leaves [6,11,46]. Previous studies on polyphenols in comfrey leaves performed by Tahirovic et al. [19] confirmed the presence of rosmarinic, caffeic and gallic acids in higher concentrations in aqueous extracts of leaves compared to roots (1.15 vs. 0.85 mg/g, 0.29 vs. 0.15 mg/g and 0.20 vs. 0.05 mg/g, respectively) [19]. In turn, Kimel et al. [11] confirmed that comfrey leaves are characterized by a lower content of pyrrolizidine alkaloids (from 6 to 14 times) compared to the roots, and their profile is also more reproducible in terms of quality and quantity, regardless of the origin of the plant material [11]. In flavonoid complexes from comfrey leaves, present in alcoholic extracts (methanol, ethanol), quercetin hexoside and quercetin acetylhexoside were detected [6]. Neagu et al. [46] detected the following flavonoids in the polyphenolic compound-rich extract of *S. officinale* leaves: rutin, luteolin, quercetin, apigenin and kaempferol [46]. In turn, in studies on flavonoids in comfrey roots using the HPLC-UV/Vis method, Paun et al. [47] demonstrated the presence of the same flavonoids in the root of Romanian comfrey, but at low concentrations: rutin (7.85 mg/kg), luteolin (1.11 mg/kg), quercetin (1.50 mg/kg), kaempferol (1.49 mg/kg) and apigenin [47].

The presented studies confirmed a statistically significant higher content of caffeic acid (0.139–0.194 mg/g dry weight) and rosmarinic acid (1.911–2.405 mg/g d.w.) in leaves compared to roots (Table 3 and Table 4). For the first time, the presence of four flavonol glycosides—quercetin and kaempferol 3-*O*-glucosides and 3-*O*-galactosides—was demonstrated in *S. officinale* leaves, and their content was determined (Table 2 and Table 4).

Two other flavonoid compounds—quercetin 3-*O*-(6″-malonyl-glucoside) (compound **5**) and kaempferol 3-*O*-(6″-malonyl-glucoside) (compound **8**)—were also identified for the first time in *S. officinale* leaves, based on their chromatographic data, namely UV spectra and ESI-MS spectra (Table 2). They were recognized only in the extract obtained by maceration (method II) (Figure 3). Previously, the presence of kaempferol hexosides, as well as quercetin O-malonyl-hexoside, was demonstrated in the aerial parts of several other *Symphytum* species [1,48,49]. Among the flavonoid compounds identified in our study, only quercetin hexoside had been previously identified in *S. officinale*, but without full characterization of the sugar moiety [6].

The observed differences in the flavonoid complexes in leaf extracts obtained by different methods indicate the influence of extraction conditions (Figure 5). The absence of both these compounds in the extract obtained by extraction in a Soxhlet apparatus may be caused by the heating temperature (aqueous bath, ~80 °C) and decomposition of both compounds. The influence of elevated temperature on this type of derivative was observed in the drying process of blackcurrant juice (*Rubus nigrum* L.)—the concentration of quercetin 3-*O*-(6″-malonyl)-glucoside in the dry extracts obtained by vacuum drying at 90 °C, they were almost twice as low (24.5–36.7 mg/kg d.w.) than using temperatures of 50 °C and 70 °C (30.9–64.4 mg/kg d.w. and 31.2–61.7 mg/kg d.w., respectively) [50]. The comparison of the chemical profiles of methanol extracts from dried comfrey leaves obtained by extraction in a Soxhlet apparatus and by maceration (room temperature) using the 2D-TLC method, confirmed the conclusion about the negative influence of the extraction conditions using the Soxhlet apparatus on the stability of malonyl esters of kaempferol and quercetin glycosides (Figure 4).

Flavonoids present in the comfrey leaves in addition to caffeic acid derivatives, may play a significant role in its antioxidant activity. They are characterized by a multidirectional mechanism of antioxidant activity, including scavenging free radicals and ROS, inhibiting the activity of enzymes involved in oxidation processes (including xanthine oxidase, nitric oxide synthase), and the ability to form complexes with metal cations. The chemical structure, particularly the presence of hydroxyl groups in the B ring and at the C-3 position (specific to flavonols), supports radical scavenging. Hydrogen bonds in the flavonoid structure stabilize the radicals formed after hydrogen donation, increasing antioxidant power [51,52].

The higher antioxidant activity of leaves compared to roots was revealed by Trifan et al. [6] for methanolic and ethanolic extracts of *S. officinale* analyzed using DPPH, FRAP, and ABTS assays. The same study showed that dichloromethane extracts were inactive. The authors indicate that the antioxidant properties of alcoholic extracts can be correlated with the presence of significant amounts of phenolic compounds, which were present in higher concentration in the leaves than in roots (total phenolics and total flavonoids). The partial least square (PLS) analysis showed that several phenolic acids, such as danshensu, dihydrogloboidnan B, rabdosiin, rosmarinic acid and dihydrogloboidnan A seemed to significantly contribute to the total antioxidant capacity, DPPH and ABTS radical scavenging activity and FRAP of *S. officinale* leaves [6].

Significant differences were demonstrated between the phytochemical profiles of the analyzed comfrey roots, both qualitatively and quantitatively, in contrast to the analyzed comfrey leaves, which were characterized by a more stable composition of active compounds, regardless of the origin of the plant raw material. In order to explain the differences between the antioxidant activity of methanol extracts from comfrey roots and leaves and the origin of the plant material—both anatomical (root/leaf) and related to the source of the raw material (botanical garden/herbal shop and geographical origin)—it was decided to conduct an in-depth chemometric analysis. Using chemometric analysis allows for the elimination of errors that may occur when comparing experimental data, including differences in chemical composition and biological activity related to growth conditions. The conducted factor analysis (FA) allowed the separation of the analyzed comfrey root samples in terms of geographical origin and cultivation conditions (producer–garden) as influencing the share of individual antioxidants in the complexes (Figure 7).

Additionally, an attempt was made to assess the anti-inflammatory activity of comfrey root and leaf extracts as well as rosmarinic acid using ready-made enzyme-linked immunosorbent assay kits (ELISA) aimed at testing the cyclooxygenase-1 and -2 (COX) inhibitory activity [COX (ovine/human) Inhibitor Screening Assay Kit]. This test directly measures the level of prostaglandin PG2α by reducing the prostaglandin PGH2 produced by the cyclooxygenase reaction with SnCl_2_ [53,54].This method has so far been successfully used to determine the anti-inflammatory activity of single isolated compounds [53,54,55,56]; however, there is only one single report on their use in the analysis of plant extracts [32]. The obtained results confirm that the mechanism of the anti-inflammatory effect of comfrey root and leaf extracts, as well as some of their ingredients, the rosmarinic acid especially, is related to the ability to inhibit the enzymatic activity of cyclooxygenase-1 and -2 [1,41,57]. Previous studies have shown that the hydroalcoholic extract, and particularly its mucilage-free fraction of comfrey root, inhibited the development of a proinflammatory state in primary human endothelial cells in a dose-dependent manner by impairing the expression of proinflammatory markers, including E-selectin, VCAM1, ICAM1, and COX-2, induced by interleukin-1 (IL-1). Both the extract and the fraction inhibited the activation of NF-κB, a transcription factor crucial for the expression of these and other proinflammatory genes [57]. Taking into account the current knowledge of the anti-inflammatory effects of hydroxycinnamates such as rosmarinic acids, it can be assumed that it considerably contributed to the COX–2 inhibitory properties of the comfrey [10,39,40,58]. The results obtained (Table 6) confirmed the anti-inflammatory effect of rosmarinic acid expressed as the ability to inhibit COX-1 and COX-2. The anti-inflammatory activity of rosmarinic acid, involving different pathways of pro-inflammatory response has been observed in vitro and in vivo—reduction of pro-inflammatory cytokines, inhibition of NFκB activation, suppression of the complement cascade, as well as the inhibitory effect on COX–2 [32,38,59,60]. Moreover, for globoidnans A and B, as constituents of another species—*Pulmonaria* sp., COX-2 inhibitory activity in silico was also revealed [32]. However, in our study, rosmarinic acid and the analyzed extracts from comfrey roots were characterized by stronger activity against COX-1, which occurs physiologically in the cells of the human body and is involved in the proper functioning of, among others, the circulatory system (inhibition of platelet aggregation) and the digestive system (including protective effects on the gastric mucosa). Inhibition of this enzyme is believed to be the primary cause of side effects associated with the use of non-selective NSAIDs (non-steroidal anti-inflammatory drugs), such as erosion and ulceration of the gastrointestinal mucosa, gastrointestinal bleeding, and impaired renal function [54]. So far, no inhibition of COX-1 activity by rosmarinic acid and root comfrey extracts has been detected. Our results are inconsistent with the literature data that showed selective COX-2 inhibitory activity of comfrey root extract [57]. The stronger inhibitory activity of rosmarinic acid and comfrey root extracts against COX-1 observed in our study may be related to the research model and test used.

In contrast to the roots, the leaves revealed stronger anti-inflammatory activity expressed primarily by inhibiting the activity of cyclooxygenase-2. This is probably the result of the very high content of flavonol compounds, quercetin and kaempferol derivatives, repeatedly described in the literature as inhibitors of cyclooxygenase-2 and -1 [61,62], as well as rosmarinic and caffeic acids [38,59,60] in the tested leaf extract. Lou et al. [10] demonstrated anti-inflammatory activity in lipopolysaccharide stimulated macrophages RAW264.7 by suppressing the expression of iNOS, COX-2, IL-1β, IL-6, TNF-α and the inactivation of NF-κB and MAPK signaling pathways for *S. officinale* leaf extract with only rosmarinic acid content determined.

There are numerous reports in the scientific literature on the cyclooxygenase-inhibiting activity (mainly against COX-2) of isoquercitrin (quercetin 3-*O*-glucoside) and astragalin (kaempferol O-glucoside)—the two main compounds dominating the flavonoid complex identified in the leaves of *S. officinale* (Table 2 and Table 4). It was revealed that isoquercetin possesses a protective effect on UVB-induced injury in cells and mice skin and it was identified as an anti-inflammatory agent by reducing the level of COX-2, and inflammatory cytokines such as IL-6, IL-1β, and TNF-α [63]. Isoquercitrin isolated from the peel of Green ball apple also decreased in a concentration-dependent manner nitric oxide (NO) production, prostaglandin E_2_, inducible NO synthase, cyclooxygenase-2 (COX-2), and nuclear factor-κB p65 protein expression. The mRNA expression of tumor necrosis factor-α, interleukin (IL)-1β, IL-6, monocyte chemoattractant protein-1, and prostaglandin E synthase 2 (PTGES2) as proinflammatory factors significantly decreased. Additionally, PTGES2, which was stimulated by COX-2 and involved in PGE_2_ expression, was inhibited [64]. In turn, in the study of nine flavonoids tested for their effects on prostaglandin E2 production and cyclooxygenase-2 expression in LPS-stimulated RAW 264.7 macrophages (apigenin, luteolin, scutellarein, quercetin, myricetin, isoquercitrin, genistein, hyperoside, rutin) all compounds effectively inhibited the LPS-induced PGE2 production but only isoquercitrin showed the significant inhibitory effect on the COX-2 expression. It was found that isoquercitrin exhibited a concentration-dependent inhibition of PGE2 production and COX-2 expression [65].

The second dominant flavonoid, astragalin, has also been shown to have anti-inflammatory activity with a multidirectional mechanism, including inhibition of COX-2 activity [66]. In the study of the anti-inflammatory properties of *Euphorbia humifusa* Willd, the n-butanol fraction was subjected to a rapid screening of candidate ligands through bio-affinity ultrafiltration with the two enzyme targets: α-glucosidase (α-Glu) and cycloxygenase-2 (COX-2) combined with UPLC/QTOF-MS. As a result, seven compounds were identified from EHNB; among them, astragalin was screened out as the one of the most active ligand compounds with specific binding affinity toward α-Glu and COX-2. The docking simulation results exhibited strong interactions of astragalin with the key residues of the enzyme targets, suggesting their possible mechanisms of action [67]. In turn, astragalin isolated from *Rosa agrestis* Savi. was investigated to estimate the anti-inflammatory effects and the underlying mechanisms on IL-1β-stimulated human osteoarthritis chondrocyte. It was found that astragalin dose-dependently inhibited IL-1β-induced NO and PGE2 production, as well as iNOS and COX-2 expression. Also, it was showed that astragalin inhibited IL-1β-induced NF-κB and MAPK activation in human osteoarthritis chondrocyte [68].

On the other hand, in a bioassay-guided in vitro screen of a 70% methanol extract of the leaves of *Salix matsudana* Koidz, which shows considerable inhibitory activity against both cyclooxygenases, it was revealed that isolated isoquercitrin exhibited moderate inhibition against COX-1 [69], while astragalin isolated from *Cornus kousa* Burg. fruits inhibited COX-1 and-2. However it should be emphasized that inhibition of COX-1 activity of isoquercitrin or astragalin is much less frequently reported [70].

Considering the stronger antioxidant activity and cyclooxygenase inhibitory activity, especially COX-2, as well as a more stable and reproducible profile of polyphenolic compounds in terms of quality/quantity, with a significantly lower content of pyrrolizidine alkaloids [11], comfrey leaves can be considered a more beneficial and safe alternative to comfrey root. The presented results provide a new insight into the anti-inflammatory activity of *Symphytum officinale*.

## 5. Conclusions

The obtained results fill the gap in the literature data regarding polyphenol profiles and the antioxidant activity of comfrey leaves and roots. Our research has shown that comfrey leaves are characterized by the presence of flavonoid glycosides and a much higher content of caffeic and rosmarinic acid compared to comfrey roots. It was confirmed that comfrey is a source of phytochemicals with antioxidant and cyclooxygenase inhibitory activity in vitro. The differences demonstrated in the levels of polyphenol content directly translate into the higher antioxidant activity of leaves, which correlated mainly with the content of caffeic acid and rosmarinic acid. The similarities demonstrated in the chemical composition of comfrey leaves and the roots of this species indicate the significant therapeutic potential of the first of the mentioned plant raw materials.

The higher content of biologically active compounds and higher antioxidant potential combined with the confirmed lower content of hepatotoxic pyrrolizidine alkaloids [7] allow comfrey leaves to be considered an alternative plant raw material to comfrey roots. However, more research needs to be conducted to assess their therapeutic effectiveness.

Chemometric analysis confirm the role of caffeic acid derivatives in the antioxidant activity of raw materials obtained from comfrey roots and leaves and allowed the separation of the analyzed samples of comfrey roots in terms of geographic origin and the cultivation conditions used (producer–garden), affecting the proportion of individual antioxidants.

## Figures and Tables

**Table 1:** Chromatographic data (t_R_, *m*/*z* M + H]^+^/[M + H]^−^, UV λ_max_) of phenolic acids and their oligomers identified in the analyzed comfrey root extract (HR1) (Kinetex F5 column). *—The identity of compound confirmed by co-chromatography with the standard.

No.	t_R_ (min)	Compound	[M + H]^+^/[M − H]^−^ [*m*/*z*]	UV λ_max_ [nm]
**1R**	9.85	Caffeic acid *	179^−^	239, 323
**2R**	22.36	Globoidnan B	539^+^/537^−^	220sh, 251, 282, 315sh, 344
**3R**	32.67	Rabdosiin	719^+^/717^−^	255sh, 282, 316sh, 344nw
**4R**	35.57	Rosmarinic acid *	361^+^/359^−^	289sh, 328
**5R**	43.57	Globoidnan A *	493^+^, 983^+^/491^−^	215, 261, 318

**Table 2:** Chromatographic data (t_R_, *m*/*z* M + H]^+^/[M + H]^−^, UV λ_max_) of phenolic compounds identified in the analyzed comfrey leaf extract HL-3 obtained by method II (maceration with methanol followed by LLE with ethyl acetate, Kinetex C18 column) (green—flavonoids, blue—phenolic acids and their oligomers).

No.	Compound Name	t_R_ [min]	UV λ_max_ [nm]	[M + H]^+^/[M − H]^−^	Mass [Da]
**1L**	Caffeic acid	8.82	295sh, 321	179^−^	180
**2L**	Quercetin 3-*O*-galactoside	25.61	254, 266sh, 300sh, 353	465^+^/463^−^	464
**3L**	Quercetin 3-*O*-glucoside	26.92	256, 266sh, 300sh, 349	465^+^/463^−^	464
**4L**	Kaempferol 3-*O*-galactoside	30.79	262, 295sh, 349	449^+^/447^−^	448
**5L**	Quercetin 3-*O*-(6″-malonyl-glucoside)	31.44	254, 266sh, 300sh, 348	551^+^	550
**6L**	Kaempferol 3*O*-glucoside	33.43	263, 296sh, 347	449^+^/447^−^	448
**7L**	Rosmarinic acid	35.11	288sh, 328	361^+^/359^−^	360
**8L**	Kaempferol 3-*O*-(6″-malonyl-glucoside)	37.63	264, 294sh, 341	535^+^, 533^−^	534
**9L**	Globoidnan A	42.33	261, 317	491^−^	492

**Table 3:** The content of caffeic acid and its derivatives (mg/g d.w. ± SD) in the analyzed samples (*n* = 3) of comfrey roots of different origin.

Comfrey Roots	Caffeic Acid (mg/g d.w. ± SD)	Globoidnan B * (mg/g d.w. ± SD)	Rosmarinic Acid (mg/g d.w. ± SD)	Globoidnan A * (mg/g d.w. ± SD)
HR1 ^a^	0.11 ± 0.004 ^egh^	0.55 ± 0.023 ^ceh^	1.18 ± 0.021 ^bcefg^	1.08 ± 0.019 ^egh^
HR2 ^b^	0.11 ± 0.003 ^egh^	0.53 ± 0.028 ^eh^	1.71 ± 0.062 ^acdeh^	0.88 ± 0.030 ^eg^
HR3 ^c^	0.11 ± 0.007 ^gh^	0.37 ± 0.101 ^afgh^	0.49 ± 0.140 ^abdfg^	0.79 ± 0.316 ^eg^
HR4 ^d^	0.11 ± 0.007 ^gh^	0.48 ± 0.021 ^gh^	1.05 ± 0.030 ^bcfg^	1.05 ± 0.005 ^egh^
GR1 ^e^	0.10 ± 0.005 ^abf^	0.34 ± 0.020 ^abfgh^	0.70 ± 0.023 ^abfg^	0.24 ± 0.006 ^abcdf^
GR2 ^f^	0.11 ± 0.003 ^egh^	0.60 ± 0.100 ^eh^	1.57 ± 0.330 ^acdeh^	0.99 ± 0.240 ^eg^
GR3 ^g^	0.09 ± 0.001 ^abcdf^	0.66 ± 0.017 ^cde^	1.80 ± 0.061 ^acdeh^	0.20 ± 0.015 ^abcdf^
GR4 ^h^	0.09 ± 0.002 ^abcdf^	0.92 ± 0.093 ^abcdefg^	0.85 ± 0.086 ^bfg^	0.59 ± 0.066 ^ad^

* Calculated on rosmarinic acid; ^abcdefgh^ Statistically significant differences in phenolic compound contents (*p* < 0.05) between analyzed plant materials; differences are indicated by the same letter (Tukey’s test).

**Table 4:** The content of phenolic acids (mg/g d.w. ± SD) and flavonoid glycosides (µg/g d.w. ± SD) in the analyzed samples (*n* = 3) of comfrey leaves of different origin.

Comfrey Leaves	Caffeic Acid (mg/g d.w. ± SD)	Rosmarinic Acid (mg/g d.w. ± SD)	Quercetin 3-*O*-Galactoside μg/g d.w. ± SD)	Quercetin 3-*O*-Glucoside (μg/g d.w. ± SD)	Kaempferol 3-*O*-Galactoside * (μg/g d.w. ± SD)	Kaempferol 3-*O*-Glucoside (μg/g d.w. ± SD)
HL1 ^a^	0.13 ± 0.01 ^c^	2.05 ± 0.16 ^c^	110.22 ± 1.59 ^c^	259.62 ± 0.81 ^ac^	100.65 ± 6.58 ^ac^	166.99 ± 3.40 ^c^
HL2 ^b^	0.14 ± 0.01 ^c^	1.91 ± 0.03 ^c^	95.62 ± 2.06 ^c^	250.58 ± 3.80 ^bc^	85.07 ± 0.75 ^bc^	148.24 ± 3.39 ^c^
HL3 ^c^	0.19 ± 0.02 ^ab^	2.41 ± 0.16 ^ab^	210.09 ± 1.30 ^ab^	959.14 ± 1.50 ^bc^	234.98 ± 11.33 ^ab^	385.94 ± 12.43 ^ab^

* Calculated on quercetin 3-*O*-galactoside (hyperoside); ^abc^ Statistically significant differences (*p* < 0.05) between analyzed plant materials; differences are indicated by the same letter (Tukey’s test).

**Table 5:** The validation data for the determination of phenolic acids and flavonoids by elaborated HPLC-UV/Vis method.

Validation Parameter		Caffeic Acid	Rosmarinic Acid	Quercetin 3-*O*-Glucoside	Kaempferol 3-*O*-Glucoside	Quercetin 3-*O*-Galactoside
Linear regression equation		y = 1641.8x − 12,817	y = 3191.4x − 12,526	y = 1596x − 1198.1	y = 1524.2x − 61.522	y = 1969.5x − 2568.1
Regression coefficient		0.9999	0.9999	0.9995	0.9998	0.9950
LOD [μg/mL]		0.122	0.3789	0.700	0.469	–
LOQ [μg/mL]		0.366	1.136	2.332	1.563	–
Recovery CV [%]	50%	–	103.56	–	–	–
	100%	–	107.25	–	–	–
	150%	–	107.82	–	–	–
Precision [CV%]	Intra-day	1.038	1.086	0.686	–	–
	Inter-day	5.12	6.82	1.030	–	–

**Table 6:** Antioxidant activity of comfrey roots and leaves of different origins using DPPH, FRAP and ABTS spectrophotometric tests (mmolTE/g ± SD) (*n* = 3).

Plant Material	DPPH (mmol TE/g ± SD)	FRAP (mmol TE/g ± SD)	ABTS (mmol TE/g ± SD)
Comfrey roots			
HR1 ^a^	0.178 ± 0.018 ^efh^	0.884 ± 0.110 ^befijk^	0.302 ± 0.024 ^efhijk^
HR2 ^b^	0.207 ± 0.002 ^defhj^	1.137 ± 0.027 ^acdeijk^	0.386 ± 0.039 ^ek^
HR3 ^c^	0.172 ± 0.007 ^efh^	0.827 ± 0.056 ^befijk^	0.292 ± 0.17 ^efhijk^
HR4 ^d^	0.168 ± 0.005 ^befh^	0.843 ± 0.016 ^befijk^	0.348 ± 0.014 ^efjk^
GR1 ^e^	0.096 ± 0.003 ^abcdgijk^	0.412 ± 0.006 ^abcdeghijk^	0.108 ± 0.003 ^abcdfghijk^
GR2 ^f^	0.097 ± 0.012 ^abcdgijk^	1.260 ± 0.037 ^acdejk^	0.471 ± 0.018 ^acdeg^
GR3 ^g^	0.203 ± 0.013 ^efhj^	1.018 ± 0.210 ^eijk^	0.364 ± 0.083 ^efjk^
GR4 ^h^	0.075 ± 0.009 ^abcdgijk^	1.031 ± 0.054 ^eijk^	0.418 ± 0.031 ^acek^
Comfrey leaves			
HL1 ^i^	0.171 ± 0.019 ^efh^	1.490 ± 0.058 ^abcdeghk^	0.427 ± 0.014 ^acek^
HL2 ^j^	0.162 ± 0.006 ^befgh^	1.520 ± 0.069 ^abcdefghk^	0.469 ± 0.025 ^acdeg^
HL3 ^k^	0.195 ± 0.025 ^efh^	1.900 ± 0.082 ^abcdefghij^	0.561 ± 0.034 ^abcdeghi^

^abcdefghijk^ Statistically significant differences in antioxidant activity expressed as mmol TE/g (*p* < 0.05) between the analyzed samples; differences are expressed by the same letter (Tukey’s test).

**Table 7:** Results of cyclooxygenase-1 and -2 inhibitory activity determinations [% inhibition ± SD] performed with the COX (ovine/human) Inhibitor Screening Assay Kit (Cayman Chemical, USA) for rosmarinic acid as a reference substance (*n* = 3).

Compound	Concentration [μM]	COX-1 [% Inhibition ± SD]	COX-2 [% Inhibition ± SD]
Rosmarinic acid	10	33.86 ± 7.96	23.16 ± 15.31
	100	42.09 ± 26.87	31.77 ± 3.63
	500	59.57 ± 4.49	36.79 ± 19.67
	IC_50_ [μM]:	300.36	1040.52

**Table 8:** Results of the determination of cyclooxygenase-1 and -2 inhibitory activity [% inhibition ± SD] performed using the COX (ovine/human) Inhibitor Screening Assay Kit (Cayman Chemical, USA) for methanol extracts Soxhlet from comfrey leaves HL3 and roots HR2 and HR3 (*n* = 3). * Result exceeding the inhibition level of the reference substance; - no result obtained.

Analyzed Methanol Extract		Concentration [mg d.w./mL]	COX-1 [% Inhibition ± SD]	COX-2 [% Inhibition ± SD]
Comfrey root	HR2	4	65.25 ± 7.01	19.63 ± 1.02
	HR3	4	55.7 ± 9.7	-
		8	*	28.86 ± 14.69
Comfrey leaf	HL3	2	-	70.93 ± 5.09
		10	33.62 ± 3.88	*

## Data Availability

The original contributions presented in this study are included in the article. Further inquiries can be directed to the corresponding author.

## References

[1] Trifan A., Wolfram E., Skalicka-Woźniak K., Luca S.V. (2024). Symphytum genus—From traditional medicine to modern uses: An update on phytochemistry, pharmacological activity, and safety. Phytochem. Rev..

[2] Staiger C. (2012). Comfrey: A clinical overview. Phytother. Res..

[3] Frost R., MacPherson H., O’Meara S. (2013). A critical scoping review of external uses of comfrey (*Symphytum* spp.). Complement. Ther. Med..

[4] Trifan A., Opitz S.E.W., Josuran R., Grubelnik A., Esslinger N., Peter S., Bräm S., Meier N., Wolfram E. (2018). Is comfrey root more than toxic pyrrolizidine alkaloids? Salvianolic acids among antioxidant polyphenols in comfrey (*Symphytum officinale* L.). roots. Food Chem. Toxicol..

[5] Nastić N., Borrás-Linares I., Lozano-Sánchez J., Švarc-Gajić J., Segura-Carretero A. (2020). Comparative Assessment of Phytochemical Profiles of Comfrey (*Symphytum officinale* L.) Root Extracts Obtained by Different Extraction Techniques. Molecules.

[6] Trifan A., Zengin G., Sinan K.I., Wolfram E., Skalicka-Woźniak K., Luca S.V. (2021). LC-HRMS/MS phytochemical profiling of *Symphytum officinale* L. and *Anchusa ochroleuca* M. Bieb. (Boraginaceae): Unveiling their multi-biological potential via an integrated approach. J. Pharm. Biomed. Anal..

[7] D’urso G., Masullo M., Seigner J., Holper-Schichl Y.M., Martin R., Plaza A., Piacente S. (2020). LC–ESI–FT–MSn metabolite profiling of symphytum officinale l. Roots leads to isolation of comfreyn a, an unusual arylnaphthalene lignan. Int. J. Mol. Sci..

[8] González D.L.N., Pérez Y.V.T., Núñez W.E.R. (2016). Determination of polyphenols and antioxidant activity of polar extracts of comfrey (*Symphytum officinale* L). Rev. Cuba. Plantas Med..

[9] Puertas-Mejía M.A., Zuleta-Montoya J.F., Rivera-Echeverry F. (2012). In vitro antioxidant capacity of comfrey (*Symphytum officinale* L.). Rev. Cuba. Plantas Med..

[10] Lou K.H., Tsai M.S., Wu J.Y. (2023). Investigating the Microwave-Assisted Extraction Conditions and Antioxidative and Anti-Inflammatory Capacities of *Symphytum officinale* WL Leaves. Processes.

[11] Kimel K., Godlewska S., Gleńsk M., Gobis K., Ośko J., Grembecka M., Krauze-Baranowska M. (2023). LC-MS/MS Evaluation of Pyrrolizidine Alkaloids Profile in Relation to Safety of Comfrey Roots and Leaves from Polish Sources. Molecules.

[12] Trifan A., Czerwińska M.E., Zengin G., Esslinger N., Grubelnik A., Wolfram E., Skalicka-Woźniak K., Luca S.V. (2023). Influence of pyrrolizidine alkaloids depletion upon the biological activity of *Symphytum officinale* L. extracts. J. Ethnopharmacol..

[13] Salehi B., Sharopov F., Boyunegmez Tumer T., Ozleyen A., Rodríguez-Pérez C., Ezzat S.M., Azzini E., Hosseinabadi T., Butnariu M., Sarac I. (2019). Symphytum Species: A Comprehensive Review on Chemical Composition, Food Applications and Phytopharmacology. Molecules.

[14] European Directorate for the Quality of Medicines & Healthcare, Council of Europe (2013). European Pharmacopoeia 8.0: Published in Accordance with the Convention on the elaboration of a European Pharmacopoeia.

[15] Kucera M., Barna M., Horácek O., Kálal J., Kucera A., Hladíkova M. (2005). Topical Symphytum herb concentrate cream against myalgia: A randomized controlled double-blind clinical study. Adv. Ther..

[16] Kučera M., Barna M., Horáček O., Kováriková J., Kučera A. (2004). Efficacy and safety of topically applied Symphytum herb extract cream in the treatment of ankle distortion: Results of a randomized controlled clinical double blind study. Wien. Med. Wochenschr.

[17] Barna M., Kucera A., Hladíkova M., Kucera M. (2012). Randomized double-blind study: Wound-healing effects of a Symphytum herb extract cream (*Symphytum×uplandicum* Nyman) in children. Arzneim. -Forsch. Drug Res..

[18] Kimel K., Zienkiewicz M., Sparzak-Stefanowska B., Krauze-Baranowska M. (2019). TLC-densitometric analysis of allantoin in *Symphytum officinale* L. roots. Acta Pharm..

[19] Tahirovic I., Rimpapa Z., Cavar S., Huseinovic S., Muradic S., Salihovic M., Sofic E. (2010). Content of some phenolic acids and rutin in the leaves and roots of *Symphytum officinale* L.. Planta Med..

[20] Sowa I., Paduch R., Strzemski M., Zielińska S., Rydzik-Strzemska E., Sawicki J., Kocjan R., Polkowski J., Matkowski A., Latalski M. (2018). Proliferative and antioxidant activity of *Symphytum officinale* root extract. Nat. Prod. Res..

[21] Pobłocka-Olech L., Krauze-Baranowska M., Godlewska S., Kimel K. (2025). HPLC-DAD-ESI/MS and 2D-TLC Analyses of Secondary Metabolites from Selected Poplar Leaves and an Evaluation of Their Antioxidant Potential. Int. J. Mol. Sci..

[22] International Conference on Harmonization of Technical Requirements for Registration of Pharmaceuticals for Human Use, ICH Harmonized Tripartite Guideline, Validation of Analytical Procedures: Text and Methodology Q2(R1); ICH Secretariat, Geneva, Switzerland, 2005. Available online: https://www.gmp-compliance.org/files/guidemgr/Q2(R1).pdf (accessed on 20 August 2023). https://www.gmp-compliance.org/files/guidemgr/Q2(R1).pdf.

[23] Jesionek A., Poblocka-Olech L., Zabiegala B., Bucinski A., Krauze-Baranowska M., Luczkiewicz M. (2018). Validated HPTLC method for determination of ledol and alloaromadendrene in the essential oil fractions of Rhododendron tomentosum plants and in vitro cultures and bioautography for their activity screening. J. Chromatogr. B Anal. Technol. Biomed. Life Sci..

[24] Bram S., Wolfram E. (2017). Recent Advances in Effect-directed Enzyme Assays based on Thin-layer Chromatography. Phytochem. Anal..

[25] Trifan A., Skalicka-Woźniak K., Granica S., Czerwińska M.E., Kruk A., Marcourt L., Wolfender J.L., Wolfram E., Esslinger N., Grubelnik A. (2020). *Symphytum officinale* L.: Liquid-liquid chromatography isolation of caffeic acid oligomers and evaluation of their influence on pro-inflammatory cytokine release in LPS-stimulated neutrophils. J. Ethnopharmacol..

[26] Ovenden S.P.B., Yu J., San Wan S., Sberna G., Murray Tait R., Rhodes D., Cox S., Coates J., Walsh N.G., Meurer-Grimes B.M. (2004). Globoidnan A: A lignan from Eucalyptus globoidea inhibits HIV integrase. Phytochemistry.

[27] (2023). COX (Ovine/Human) Inhibitor Screening Assay Kit Booklet.

[28] Okamura K.M., Hibi K., Nakamura H. (1990). A Calculation Method of Correspoding Mobile Phase Composition for Obtaining the Same Retention with Different Binary Organic/Aqueous Solvent Systems in Reversed Phase Liquid Chromatography. Anal. Sci..

[29] Markham K.R. (1982). Techniques of Flavonoids Identification.

[30] Shekarchi M., Hajimehdipoor H., Saeidnia S., Gohari A.R., Hamedani M.P. (2012). Comparative study of rosmarinic acid content in some plants of Labiatae family. Pharmacogn. Mag..

[31] Tufa T., Damianakos H., Zengin G., Graikou K., Chinou I. (2019). Antioxidant and enzyme inhibitory activities of disodium rabdosiin isolated from Alkanna sfikasiana Tan, Vold and Strid. S. Afr. J. Bot..

[32] Krzyżanowska-Kowalczyk J., Kowalczyk M., Ponczek M.B., Pecio Ł., Nowak P., Kolodziejczyk-Czepas J. (2021). Pulmonaria obscura and pulmonaria officinalis extracts as mitigators of peroxynitrite-induced oxidative stress and cyclooxygenase-2 inhibitors–in vitro and in silico studies. Molecules.

[33] Choma I.M., Nikolaichuk H. (2022). TLC bioprofiling—A tool for quality evaluation of medicinal plants. Evidence-Based Validation of Herbal Medicine: Translational Research on Botanicals.

[34] Nikolaichuk H., Studziński M., Choma I.M. (2020). Effect directed detection of *Rhodiola rosea* L. root and rhizome extract. J. Liq. Chromatogr. Relat. Technol..

[35] Ciesla Ł.M., Waksmundzka-Hajnos M., Wojtunik K.A., Hajnos M. (2015). Thin-layer chromatography coupled with biological detection to screen natural mixtures for potential drug leads. Phytochem. Lett..

[36] Vuolo M.M., Lima V.S., Maróstica Junior M.R., Campos M.R.S. (2019). Chapter 2—Phenolic Compounds: Structure, Classification, and Antioxidant Power. Bioactive Compounds.

[37] Fujimoto A., Masuda T. (2012). Antioxidation mechanism of rosmarinic acid, identification of an unstable quinone derivative by the addition of odourless thiol. Food Chem..

[38] Colica C., Di Renzo L., Aiello V., De Lorenzo A., Abenavoli L. (2018). Rosmarinic acid as potential anti-inflammatory agent. Rev. Recent Clin. Trials.

[39] Kim G.D., Park Y.S., Jin Y.H., Park C.S. (2015). Production and applications of rosmarinic acid and structurally related compounds. Appl. Microbiol. Biotechnol..

[40] Bulgakov V.P., Inyushkina Y.V., Fedoreyev S.A. (2012). Rosmarinic acid and its derivatives: Biotechnology and applications. Crit. Rev. Biotechnol..

[41] Mahmoudzadeh E., Nazemiyeh H., Hamedeyazdan S. (2022). Anti-inflammatory Properties of the Genus *Symphytum* L.: A Review. Iran. J. Pharm. Res..

[42] Alizadeh P., Rahimi M., Amjadi S., Bayati M., Nejad Ebrahimi S. (2024). Enrichment of rosmarinic acid from comfrey (*Symphytum officinale* L.) root extract by macroporous adsorption resins and molecular docking studies. Ind. Crops Prod..

[43] Savić V.L., Savić S.R., Nikolić V.D., Nikolić L.B., Najman S.J., Lazarević J.S., Đorđević A.S. (2015). The identification and quantification of bioactive compounds from the aqueous extract of comfrey root by UHPLC–DAD–HESI–MS method and its microbial activity. Hem. Ind..

[44] Trifan A., Wolfram E., Esslinger N., Grubelnik A., Skalicka-Woźniak K., Minceva M., Luca S.V. (2021). Globoidnan A, rabdosiin and globoidnan B as new phenolic markers in European-sourced comfrey (*Symphytum officinale* L.) root samples. Phytochem. Anal..

[45] Trifan A., Zengin G., Sinan K.I., Esslinger N., Grubelnik A., Wolfram E., Skalicka-woźniak K., Minceva M., Luca S.V. (2021). Influence of the post-harvest storage time on the multi-biological potential, phenolic and pyrrolizidine alkaloid content of comfrey (*Symphytum officinale* L.) roots collected from different european regions. Plants.

[46] Neagu E., Paun G., Albu C., Eremia S.A., Radu G.L. (2023). Artemisia abrotanum and *Symphytum officinale* Polyphenolic Compounds-Rich Extracts with Potential Application in Diabetes Management. Metabolites.

[47] Paun G., Neagu E., Litescu S.C., Rotinberg P., Radu G.L. (2012). Application of membrane processes for the concentration of *Symphytum officinale* and *Geranium robertianum* extracts to obtain compounds with high anti-oxidative activity. J. Serbian Chem. Soc..

[48] Luca S.V., Zengin G., Kulinowski Ł., Sinan K.I., Skalicka-Woźniak K., Trifan A. (2024). Phytochemical profiling and bioactivity assessment of underutilized Symphytum species in comparison with *Symphytum officinale*. J. Sci. Food Agric..

[49] Varvouni E.-F., Zengin G., Graikou K., Ganos C., Mroczek T., Chinou I. (2020). Phytochemical analysis and biological evaluation of the aerial parts from *Symphytum anatolicum* Boiss. and *Cynoglottis barrelieri* (All.) Vural & Kit Tan (Boraginaceae). Biochem. Syst. Ecol..

[50] Michalska A., Wojdyło A., Brzezowska J., Majerska J., Ciska E. (2019). The influence of inulin on the retention of polyphenolic compounds during the drying of blackcurrant juice. Molecules.

[51] Shen N., Wang T., Gan Q., Liu S., Wang L., Jin B. (2022). Plant flavonoids: Classification, distribution, biosynthesis, and antioxidant activity. Food Chem..

[52] Yang B., Liu H., Yang J., Gupta V.K., Jiang Y. (2018). New insights on bioactivities and biosynthesis of flavonoid glycosides. Trends Food Sci. Technol..

[53] Gorzkiewicz M., Janaszewska A., Ficker M., Svenningsen S.W., Christensen J.B., Klajnert-Maculewicz B. (2019). Pyrrolidone-modified PAMAM dendrimers enhance anti-inflammatory potential of indomethacin in vitro. Colloids Surf. B Biointerfaces.

[54] Fernandes C., Palmeira A., Ramos I.I., Carneiro C., Afonso C., Tiritan M.E., Cidade H., Pinto P.C.A.G., Saraiva M.L.M.F.S., Reis S. (2017). Chiral derivatives of xanthones: Investigation of the effect of enantioselectivity on inhibition of cyclooxygenases (COX-1 and COX-2) and binding interaction with human serum albumin. Pharmaceuticals.

[55] Killari K.N., Thuan N.H., Prasanth D., Panda S.P., Pasala P.K., Ketha A., Tatipamula V.B. (2023). Bioassay Guided Isolation of Anti-Inflammatory Compounds from *Bauhinia variegata* L.: A Key Ingredient in Herbo-Mineral Formulation, Gandmala Kandan Ras. Indian J. Pharm. Sci..

[56] Nguyen H.T., Vu T.Y., Chandi V., Polimati H., Tatipamula V.B. (2020). Dual COX and 5-LOX inhibition by clerodane diterpenes from seeds of *Polyalthia longifolia* (Sonn.) Thwaites. Sci. Rep..

[57] Seigner J., Junker-Samek M., Plaza A., D’Urso G., Masullo M., Piacente S., Holper-Schichl Y.M., De Martin R. (2019). A symphytum officinale root extract exerts anti-inflammatory properties by affecting two distinct steps of NF-κB signaling. Front. Pharmacol..

[58] Marchev A.S., Vasileva L.V., Amirova K.M., Savova M.S., Koycheva I.K., Balcheva-Sivenova Z.P., Vasileva S.M., Georgiev M.I. (2021). Rosmarinic acid—From bench to valuable applications in food industry. Trends Food Sci. Technol..

[59] Chockalingam N., Muruhan S. (2017). Anti-inflammatory properties of rosmarinic acid—A review. Int. J. Res. Pharm. Sci..

[60] Luo C., Zou L., Sun H., Peng J., Gao C., Bao L., Ji R., Jin Y., Sun S. (2020). A Review of the Anti-Inflammatory Effects of Rosmarinic Acid on Inflammatory Diseases. Front. Pharmacol..

[61] Lee G.B., Kim Y., Lee K.E., Vinayagam R., Singh M., Kang S.G. (2024). Anti-Inflammatory Effects of Quercetin, Rutin, and Troxerutin Result From the Inhibition of NO Production and the Reduction of COX-2 Levels in RAW 264.7 Cells Treated with LPS. Appl. Biochem. Biotechnol..

[62] Devi K.P., Malar D.S., Nabavi S.F., Sureda A., Xiao J., Nabavi S.M., Daglia M. (2015). Kaempferol and inflammation: From chemistry to medicine. Pharmacol. Res..

[63] Li Y., Ma Y., Yao Y., Ru G., Lan C., Li L., Huang T. (2024). Protective effect of isoquercitrin on UVB-induced injury in HaCaT cells and mice skin through anti-inflammatory, antioxidant, and regulation of MAPK and JAK2-STAT3 pathways. Photochem. Photobiol..

[64] Lee E.H., Park H.J., Jung H.Y., Kang I.K., Kim B.O., Cho Y.J. (2022). Isoquercitrin isolated from newly bred Green ball apple peel in lipopolysaccharide-stimulated macrophage regulates NF-κB inflammatory pathways and cytokines. 3 Biotech.

[65] Xu Y., Wang C., Wu L., Li Z., Chen M., Wang Y., Li F., Luo C. Comparison of inhibitory effects of nine flavonoids on prostaglandin E 2 production and COX-2 expression in LPS-stimulated RAW264.7 macrophages. Proceedings of the 2012 International Conference on Biomedical Engineering and Biotechnology, iCBEB 2012.

[66] Riaz A., Rasul A., Hussain G., Zahoor M.K., Jabeen F., Subhani Z., Younis T., Ali M., Sarfraz I., Selamoglu Z. (2018). Astragalin: A Bioactive Phytochemical with Potential Therapeutic Activities. Adv. Pharmacol. Sci..

[67] Rakotondrabe T.F., Fan M., Guo M. (2022). Exploring potential antidiabetic and anti-inflammatory flavonoids from Euphorbia humifusa with an integrated strategy. Front. Pharmacol..

[68] Ma Z., Piao T., Wang Y., Liu J. (2015). Astragalin inhibits IL-1β-induced inflammatory mediators production in human osteoarthritis chondrocyte by inhibiting NF-κB and MAPK activation. Int. Immunopharmacol..

[69] Li X., Liu Z., Zhang X.F., Wang L.J., Zheng Y.N., Yuan C.C., Sun G.Z. (2008). Isolation and characterization of phenolic compounds from the leaves of Salix matsudana. Molecules.

[70] Vareed S.K., Schutzki R.E., Nair M.G. (2007). Lipid peroxidation, cyclooxygenase enzyme and tumor cell proliferation inhibitory compounds in Cornus kousa fruits. Phytomedicine.

